# Semantic segmentation of plant roots from RGB (mini-) rhizotron images—generalisation potential and false positives of established methods and advanced deep-learning models

**DOI:** 10.1186/s13007-023-01101-2

**Published:** 2023-11-06

**Authors:** Pavel Baykalov, Bart Bussmann, Richard Nair, Abraham George Smith, Gernot Bodner, Ofer Hadar, Naftali Lazarovitch, Boris Rewald

**Affiliations:** 1 Institute of Forest Ecology, Department of Forest and Soil Sciences, University of Natural Resources and Life Sciences, Vienna (BOKU), Vienna, Austria; 2 Vienna Scientific Instruments GmbH, Alland, Austria; 3 IDLab, Department of Computer Science, University of Antwerp - Imec, Antwerp, Belgium; 4 Dept. Biogeochemical Integration, Max Planck Institute for Biogeochemistry, Jena, Germany; 5 Department of Computer Science, University of Copenhagen, Copenhagen, Denmark; 6 Institute of Agronomy, University of Natural Resources and Life Sciences, Vienna (BOKU), Vienna, Austria; 7 School of Electrical and Computer Engineering, Ben-Gurion University of the Negev, Beer Sheva, Israel; 8 Wyler Department for Dryland Agriculture, French Associates Institute for Agriculture and Biotechnology of Drylands, Jacob Blaustein Institutes for Desert Research, Ben-Gurion University of the Negev, Sede Boqer Campus, Beersheba, Israel; 9 Faculty of Forestry and Wood Technology, Mendel University in Brno, Brno, Czech Republic; 10 Discipline of Botany, School of Natural Sciences, Trinity College, Dublin, Ireland

**Keywords:** Automatic image segmentation, Data augmentation, Deep learning, False positives, Fine roots, Image processing, Minirhizotron, Neural networks, Root segmentation, U-Net

## Abstract

**Background:**

Manual analysis of (mini-)rhizotron (MR) images is tedious. Several methods have been proposed for semantic root segmentation based on homogeneous, single-source MR datasets. Recent advances in deep learning (DL) have enabled automated feature extraction, but comparisons of segmentation accuracy, false positives and transferability are virtually lacking. Here we compare six state-of-the-art methods and propose two improved DL models for semantic root segmentation using a large MR dataset with and without augmented data. We determine the performance of the methods on a homogeneous maize dataset, and a mixed dataset of > 8 species (mixtures), 6 soil types and 4 imaging systems. The generalisation potential of the derived DL models is determined on a distinct, unseen dataset.

**Results:**

The best performance was achieved by the U-Net models; the more complex the encoder the better the accuracy and generalisation of the model. The heterogeneous mixed MR dataset was a particularly challenging for the non-U-Net techniques. Data augmentation enhanced model performance. We demonstrated the improved performance of deep meta-architectures and feature extractors, and a reduction in the number of false positives.

**Conclusions:**

Although correction factors are still required to match human labelled root lengths, neural network architectures greatly reduce the time required to compute the root length. The more complex architectures illustrate how future improvements in root segmentation within MR images can be achieved, particularly reaching higher segmentation accuracies and model generalisation when analysing real-world datasets with artefacts—limiting the need for model retraining.

**Supplementary Information:**

The online version contains supplementary material available at 10.1186/s13007-023-01101-2.

## Background

Despite a recent increase in research on plant roots, studies addressing ecosystem processes below ground and underlying (root) traits are still relatively rare compared to aboveground measurements. However, roots play a key role in plant function (e.g., water and nutrient uptake, anchorage, propagation) and affect many ecosystem processes such as chemical transformation and circulation of substances from the atmosphere to the lithosphere, and particularly the formation and stabilisation of soil organic matter [1–3]. In specific, information on root system development in time and space is critical to determine plants’ resource allocation patterns [4–6] and the soil volume explored for resource exploitation [7, 8].

**Fig. 1:**
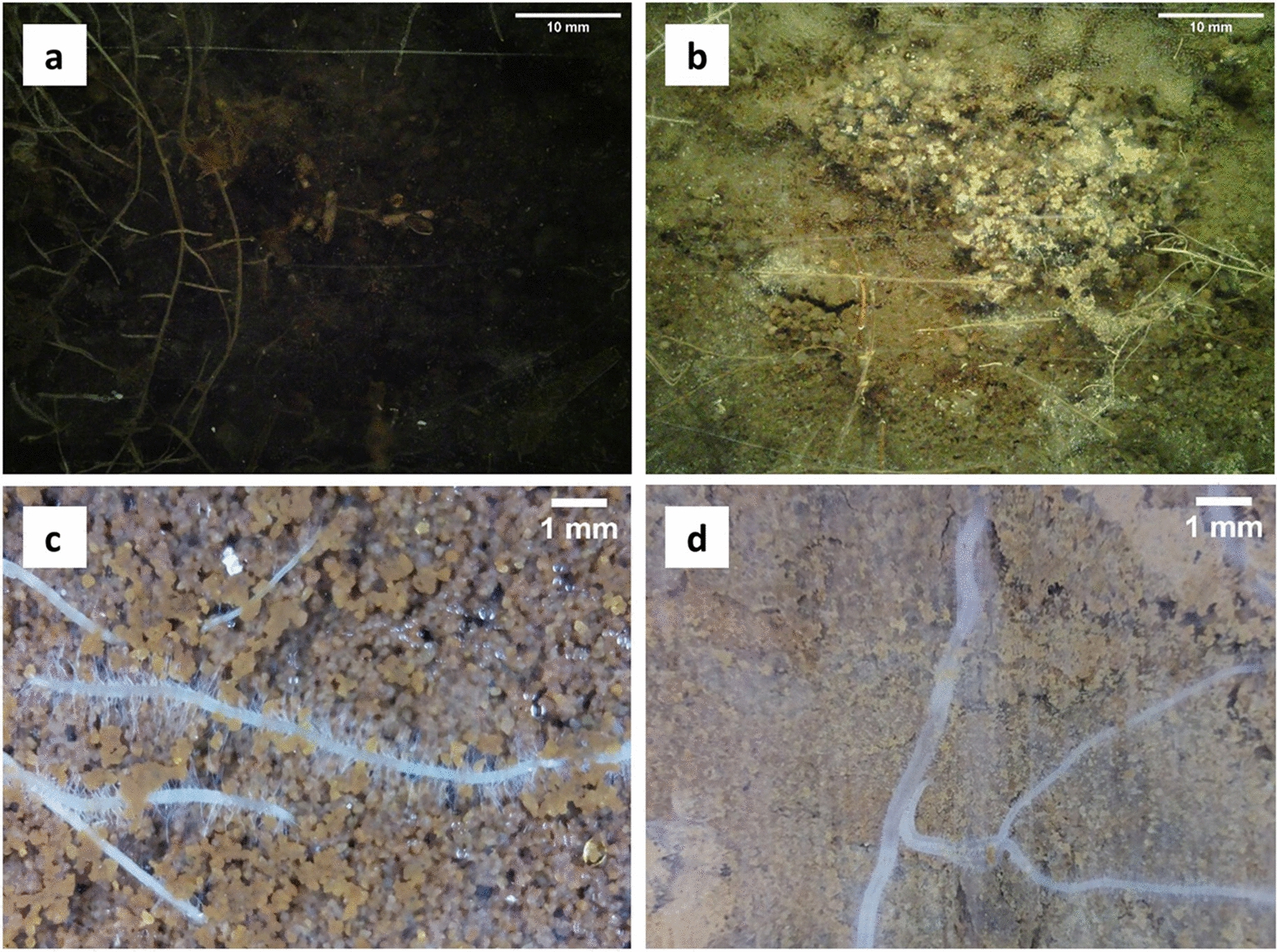
Example minirhizotron images illustrating the varying image qualities and properties used in this study. **a** and **b** are images (tree-grassland ecosystem) of the MANIP project dataset; **c** and **d** are maize (*Zea mays*) and olive (*Olea europaea*) roots from the ATTRACT project dataset, respectively. Both datasets are using different cameras system, focus, resolution, and illumination (see Method section for details on projects/datasets). Subpanels **a** and **b** present heterogeneous lighting but overall dark images with a lot of shadows. Both **c** and **d** exhibit uniform lighting without significant shadows, but there are distinct variations in soil moisture levels on the outer surface of the MR tubes

Beyond the traditional destructive soil sampling and root washing, and with advances in (imaging) technology, root studies have gradually become easier—allowing significant advances in root phenotyping [9]. Root observation methods in soil, allowing for repeated measurements, range from X-ray tomography [10] to Electrical Resistance Tomography [11]. Among the diverse non-destructive techniques (mini-) rhizotrons (MR), or rhizoboxes, allow (periodic) collection of 2D images of roots growing adjacent to a transparent tube or plane “window”. Rhizotron systems with flat, transparent (plexi-)glass windows allow taking larger but structurally similar images than MR cameras (in tubes), at the cost of higher infrastructure costs, potentially greater (soil) disturbance and a less flexible use for experiments. There is a consensus in the community that MR camera systems are currently the best approach for observing the timing (phenology) of root emergence, growth and decay (turnover) under field conditions—providing a 'window' into a relatively undisturbed rhizosphere that most other methods do not [12]. Acrylic MR tubes are the least likely to affect root characteristics [13], however, being easily scratched during installation may already affect image quality. In addition, a variety of conditions (e.g., large gaps/voids between the MR tube and the soil) may affect root growth patterns and the quality of the images/results obtained, or at least impact the analysis. The most common data produced by CCD- or CMOS-type (i.e., scanner- or camera-type) (mini-)rhizotrons are RGB images, while trials with multispectral MR cameras have started more recently [14, 15]. Due to the variety of commercial MR imaging devices (Additional file 1) and "do it yourself" solutions [16], different sensors with distinct resolution, focus, and illumination produce very different image qualities (Fig. 1)., In addition, smartphones have recently been suggested as effective devices for image acquisition in rhizotron and “root window" settings [17].

Analysis of MR images is still a challenge, and software such as RootFly [18] or rhizoTrak [19], allow calculation of root lengths and widths, but require the user to (semi) manually trace roots on the image. As manual root identification can be very tedious, time-consuming and requires trained annotators, (semi-)automatic software tools are in high demand. All of them start in one way or another with semantic segmentation or identification of roots, or related parts such as nodules [20], from the RGB image. Tools such as DART [21], SmartRoot [22], EZ-Rhizo [23], DIRT [24], GiA-Roots [25], RootNav [26], GT-RootS [27] generally perform very well in detecting and analysing root systems and traits in the target environments for which they were developed, but their transferability is often questioned. In particular, they are often reliable at segmenting roots with high contrast to a more homogeneous background [9]. In contrast, images with heterogeneous backgrounds that contain artefacts (e.g., petioles or plastic foils) and roots of different shapes and colours (some partially hidden in the soil), can be challenging.

Subsequently, more recent approaches have already included techniques allowing for semi-automated root detection in more heterogeneous soil conditions. Adaptive thresholding, used by saRIA [28], differs from global thresholding by taking into account spatial variations in illumination [29]. Frangi Vesselness [30], a filtering technique capable of recognising tubular structures [31], has also been applied to root segmentation tasks [32, 33]. Yu et al. [34] achieved root segmentation using a multi-instance support vector machine (SVM). SVM is an algorithm that maps the data into a feature space (i.e., a multidimensional space) in which a hyperplane with a maximum margin of separation between two classes is optimised [35]. However, it is the development of deep learning (DL) techniques [36] that has opened up the possibility of automated root segmentation in real-world conditions. In particular, convolutional neural networks (CNN) are considered an effective method that combines DL and computer vision technology to extract target features directly from an input image [37]. While CNN architectures have been explored for root image analyses a decade ago [26], Wang et al. [38] only recently reported a fully automatic, DL-based feature extraction method—SegRoot, an artificial neural network model with a simple DL architecture of encoder-decoder. A more recent study using the U-Net model [33] also provided good segmentation, leading to the development of the versatile RootPainter tool [20]. However, trained DL models may not perform similarly under different species × soil types, or when using different imaging devices, if this was not taken into account during training. Ward and Moghadam [39] recently showed that the background texture in particular has a significant effect on leaf segmentation performance; soil backgrounds are expected to be even more heterogeneous. One way, as implemented in RootPainter, is to make retraining on a target dataset fast and accessible using an interactive ML process that involves simultaneous annotation and training, connected in a real-time feedback loop [17, 20]. However, to largely avoid re-training a DL model can also be trained on a diverse dataset to generalise root segmentation, so that the model learns to recognise most types of roots under different soil and imaging conditions. For reliable monitoring of the spatial distribution of root, it is essential that “false positives”, such as the segmentation of roots in "soil only" images, are limited.

Building on these recent advances, here we develop an improved approach to root segmentation based on DL and specifically U-Net architecture: a U-Net backboned with either EfficientNet [40] or with SE-ResNeXt-101 (32 × 4d) [41] as encoders. Both architectures have already shown impressive results in image recognition tasks [40, 42, 43]. A backboned model is a neural network architecture [either EfficientNet or SE-ResNeXt-101 (32 × 4d)] that serves as the main feature extractor in a much larger architecture (U-Net). A basic, default U-Net architecture consists of contracting and expansive paths [44], i.e. encoder and decoder; both of which can be modified in order to achieve better performance. Different architectures can be used as the encoder or the decoder backbone, e.g., U-Net + + with the EfficientNet backbone [43, 45, 46]. As data augmentation is considered a powerful method to reduce training errors and overfitting of DL models [39, 47], it can be used to extend regular data training (i.e. original images + masks)—particular when time-consuming masking limits data availability.

Therefore, the aim of this study is on the one hand, to compare established techniques for root segmentation, i.e., image processing techniques (Frangi Vesselness, adaptive thresholding), machine learning algorithm (SVM) and deep learning segmentation models (SegRoot, basic U-Net) with a novel approach based on backboned U-Net. On the other hand, we aim to determine the generalisation potential of these techniques/models by training on a mixed real-world dataset (> 6 species, 4 soil types, and 3 imaging devices) and applying the models to an unseen, distinct (in terms of species, soil type and imaging device) dataset.

## Results and discussion

### Simple maize data

All applied methodologies segmented roots in validation images of *Zea mays* (Table 1, Additional file 4)—illustrating the general applicability but varying performance of the techniques on a “simple” MR data set—i.e. holding a good contrast between white *Zea mays* roots and darker, homogeneous sandy soil with limited artefacts (Fig. 1c).

**Table 1:** Performance of different techniques/models (see Table 4 for details) on the homogeneous *Zea mays* (ATTRACT 1) validation set

Technique/model	SSIM	DSC	IoU
Dummy classifier	0.9154	0.0032	0.0032
Frangi Vesselness	0.4222	0.2634	0.1639
Adaptive thresholding	0.8076	0.5033	0.3664
SVM	0.8326	0.5804	0.4271
SegRoot	0.9203	0.4460	0.3122
UNetGNRes	0.9498	0.6708	0.5380
U-Net SE-ResNeXt-101 (32 × 4d)	0.9534	0.6968	0.5607
U-Net EfficientNet-b6	**0.9551**	**0.7213**	**0.5860**

The best scores for the evaluation metrics average structural similarity index (SSIM), average Sørensen–Dice similarity coefficient (DSC), and average Jaccard index/Intersection over Union (IoU) are given in bold

**Fig. 2:**
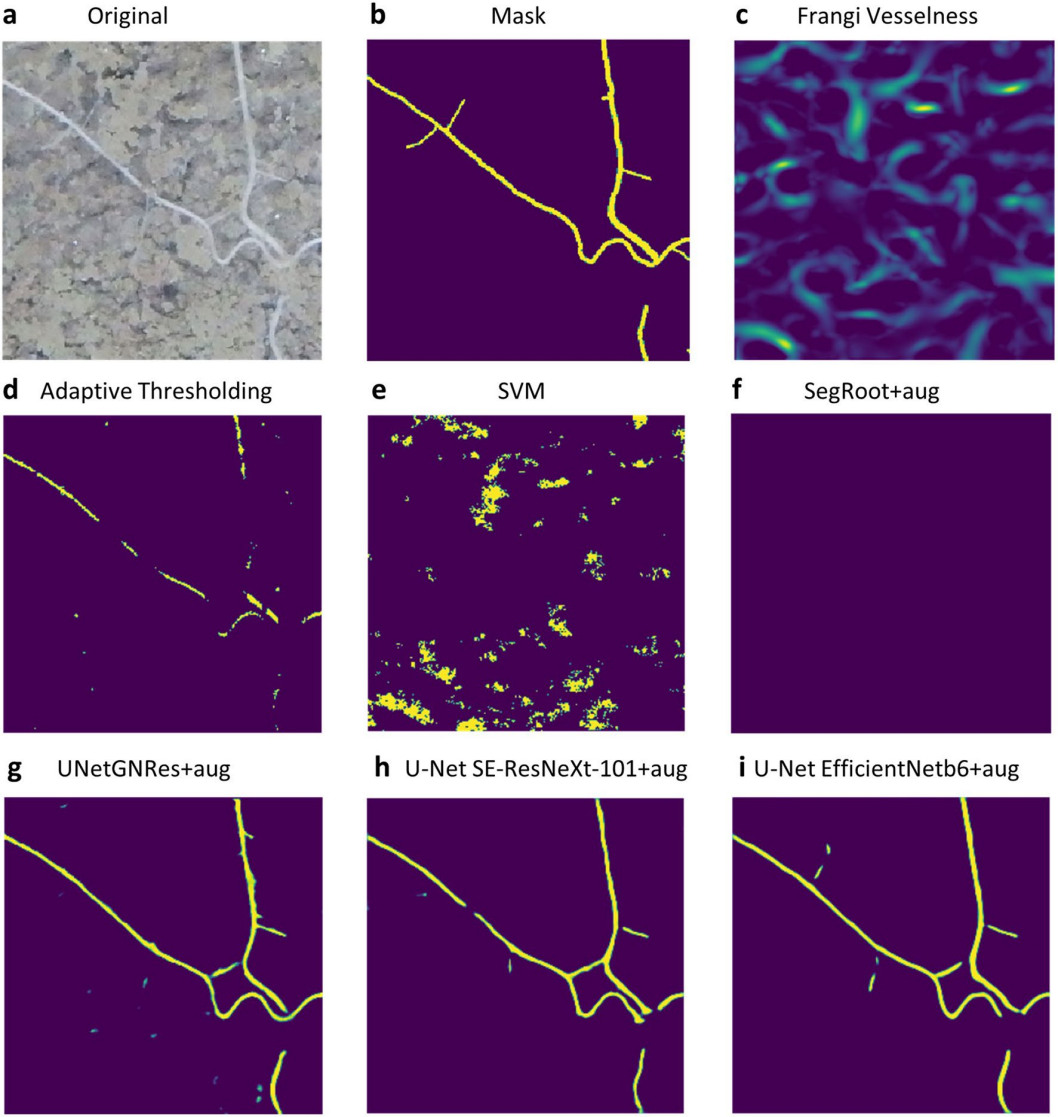
Masks of prediction examples on the ‘unseen’ test data of the *Cichorium intybus* (RootPainter) dataset; models e-i were trained on the Mixed dataset. Original image from a rhizotron **a**, manually labelled mask **b**, and masks derived using the techniques/models: Frangi Vesselness **c**, Adaptive thresholding **d**, Support Vector Machine (SVM) **e**, SegRoot **f**, UNetGNRes **g**, U-Net SE-ResNeXt-101 (32 × 4d) **h**, and U-Net EfficientNet-b6 **i**. Only the best DL models (**f–i**; Table 3), i.e., trained with augmented data (+ aug), are displayed; see Table 4, Additional file 2 and text for details

The SSIM metric, which indicates the image similarity index between the manual and the predicted mask, is extremely high (~ 0.92) for the same label predictor, which can be explained by the fact that most of the pixels in the mask are “(soil) background” and not roots. In contrast, DSC and IoU are metrics that indicate segmentation accuracy; the IoU is typically lower than DSC, because IoU penalises bad segmentations to a greater extent. However, both are strongly correlated (Tables 1–3); in the case of the dummy classifier, DSC and IoU have equal scores and low values because there are no “soil only” images in the *Zea mays* validation set. For Frangi Vesselness, adaptive thresholding and SVM, average DSCs were higher and SSIMs were lower compared to the dummy classifier (Table 1). This may indicate that the predicted masks contain many pixels that are incorrectly classified as root (as illustrated in Fig. 2). In particular, Frangi Vesselness shows complex images (Fig. 2c) with a high diversity of pixel values between 0 and 1. This results in “blurred” predicted masks with a large numbers of tubular structures (Fig. 2c, Additional file 4).

Within the DL models, SegRoot is outperformed by both the U-Net models, SVM and even adaptive thresholding, based on IoU or DSC indices. Within the U-Net architectures presented here, backboned U-Net architectures have higher average SSIM, DSC and IoU compared to the native encoder of UNetGNRes. All DL models compared here have a similar architecture: an encoder and a decoder, joined by a bottleneck. Based on the encoder-decoder concept many other architectures have been derived, such as SegNet [48] or U-Net [44],which additionally include some skipping connections—“skipping” the bottleneck. SegRoot for instance is a modification of SegNet [38]. The SE-ResNeXt-101 (32 × 4d) architecture, which acts as an encoder here, has previously demonstrated high accuracies with a relatively small number of operations in image recognition tasks [42]. Alternatively, EfficientNet is a more recent architecture for image recognition that has also shown remarkably high accuracies with “fewer” parameters (43 million, Additional file 3) compared to architectures such as SENet (146 million parameters) [40]. Thus, the combination of powerful encoders with a segmentation architecture such as U-Net may underlie the higher performances observed (Table 1) and reported previously [45, 46].

Adaptive thresholding has earlier shown satisfactory results for root segmentation and posterior root length calculation, with a mean DSC of 0.82 and an R^2^ of 0.849 for the regression of predicted *vs.* human-labelled length [28]. However, here, the application of the technique on high quality images of *Zea mays* roots resulted only in a moderate DSC of 0.50; considering that the best model showed a DSC of 0.72 (Table 1). This highlights the challenge of identifying roots when high quality and close focused images are available; the presence of a few white and bright artefacts (mostly little rocks and water drops, but also mycelium) can severely restrict root segmentation. Another possible explanation regarding the low DSC and IoU may, however, be the reduced size of the images which lost information that could be valuable for some methods such as artificial neural networks [49].

### Mixed dataset composed of different species, soils and artefacts

The *Zea mays* dataset discussed above (Table 1) represents a situation as commonly used for method development in automating root segmentation, i.e., one species in one soil type [33, 37, 38]. However, the here compiled mixed dataset represents a more challenging situation—containing images with various types of roots in different soils and artefacts (Additional file 4). A complex dataset will reveal the generalisation potential of the methods; however, to the best of our knowledge, no similar attempts have been published yet. Here, all methods were evaluated on a “test” subset of the mixed dataset (Table 2).

**Table 2:** Performance of different techniques/models (Table 4) on a test data subset of the mixed data set, which was not used during the training on the mixed data set

Technique/model (+ aug)	SSIM	DSC	IoU	FPR
Dummy classifier^b^	0.9173	0.3250	0.3250	-
Frangi Vesselness	0.3665	0.1009	0.0626	1.0
Adaptive thresholding	0.8348	0.2367	0.1804	0.8636
SVM	0.7617	0.1744	0.1341	0.9090
SegRoot^ab^	0.9173	0.3250	0.3250	-
SegRoot + aug^ab^	0.9173	0.3250	0.3250	-
UNetGNRes	0.9246	0.4399	0.3585	0.6363
UNetGNRes + aug	0.9313	0.5326	0.4452	0.4545
U-Net SE-ResNeXt-101 (32 × 4d)	0.9352	0.5708	0.4800	0.3636
U-Net SE-ResNeXt-101 (32 × 4d) + aug	0.9360	0.6217	0.5299	0.2272
U-Net EfficientNet-b6	0.9375	0.6418	0.5498	0.1363
U-Net EfficientNet-b6 + aug	**0.9381**	**0.6848**	**0.5920**	**0.0454**

The best scores for the evaluation metrics average structural similarity index (SSIM), average Sørensen-Dice similarity coefficient (DSC), and average Jaccard index/intersection over union (IoU), and the lowest false positive rate (FPR) are shown in bold; Number of images used: Training: 557/augmented Training: 2228, Validation: 62, Testing: 69. Models trained with augmented data are indicated by + aug

^a^No differences in model performance were found using + aug in the SegRoot model

^b^EXCLUDED from FPR determination as not predicting roots. indicate methods where the labels are zero or do not predict roots

**Fig. 3:**
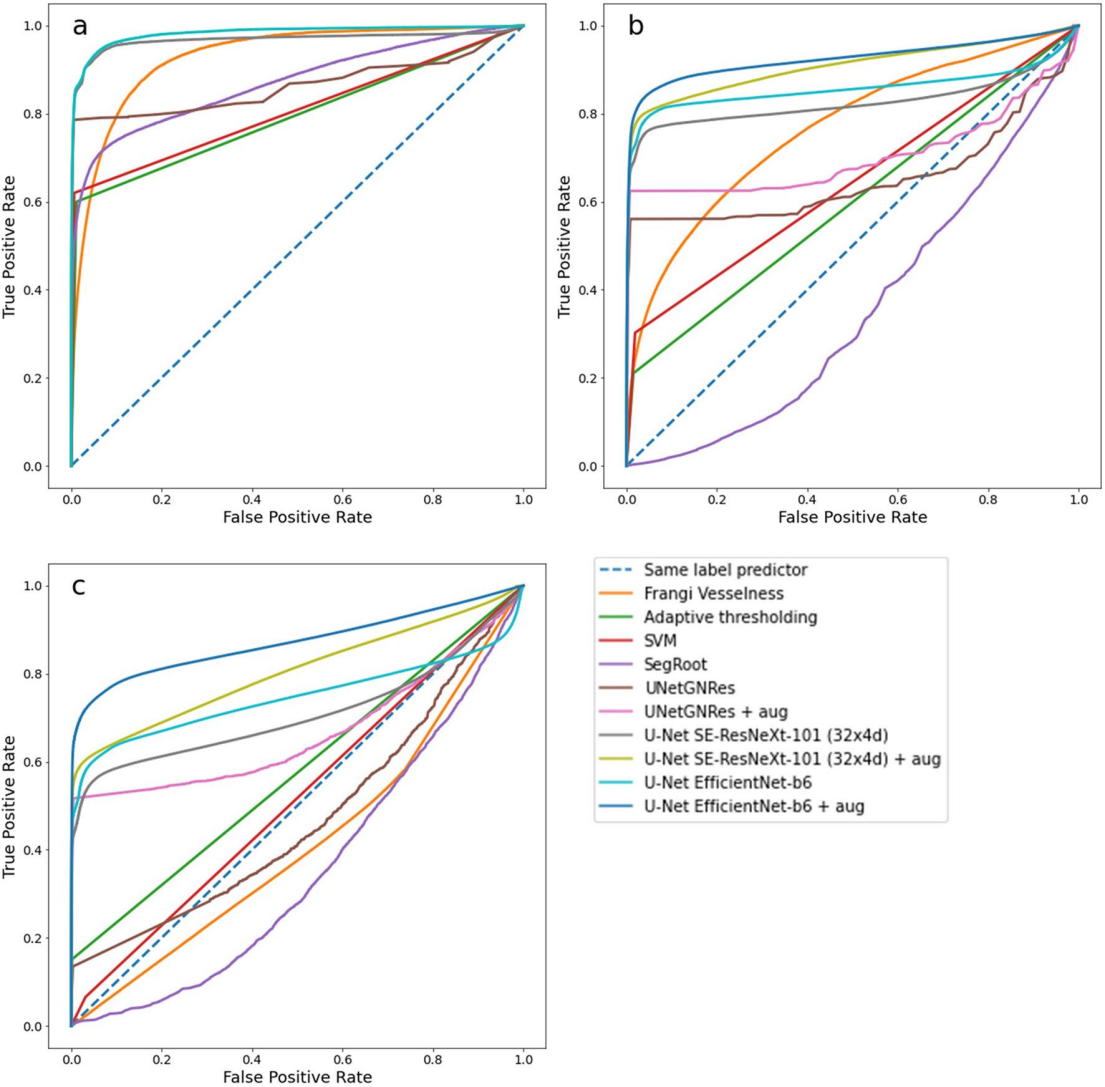
Receiver operating characteristic (ROC) curves of true vs false positive rates (FPR) on MR images of **a** training data set of *Zea mays* roots (“ATTRACT 1” project), without augmentation, **b** the mixed validation data set with augmentation (+ aug), and **c** the unseen *Cichorium intybus* (RootPainter) data set (Additional file 2). A diagonal dashed line indicates the dummy classifier, values above the line are better, values below are worse than a random classifier. The “elbow” on the left indicates a more “conservative” classifier, such as Adaptive Thresholding, while being on the right indicates a more “liberal” classifier, such as Frangi Vesselness. The closer the “elbow” is to the upper left corner (0,1), the better the model. ROCs of all methods are shown in different colours; SegRoot + aug is not shown for clarity (it largely overlaps with the dummy classifier)

**Fig. 4:**
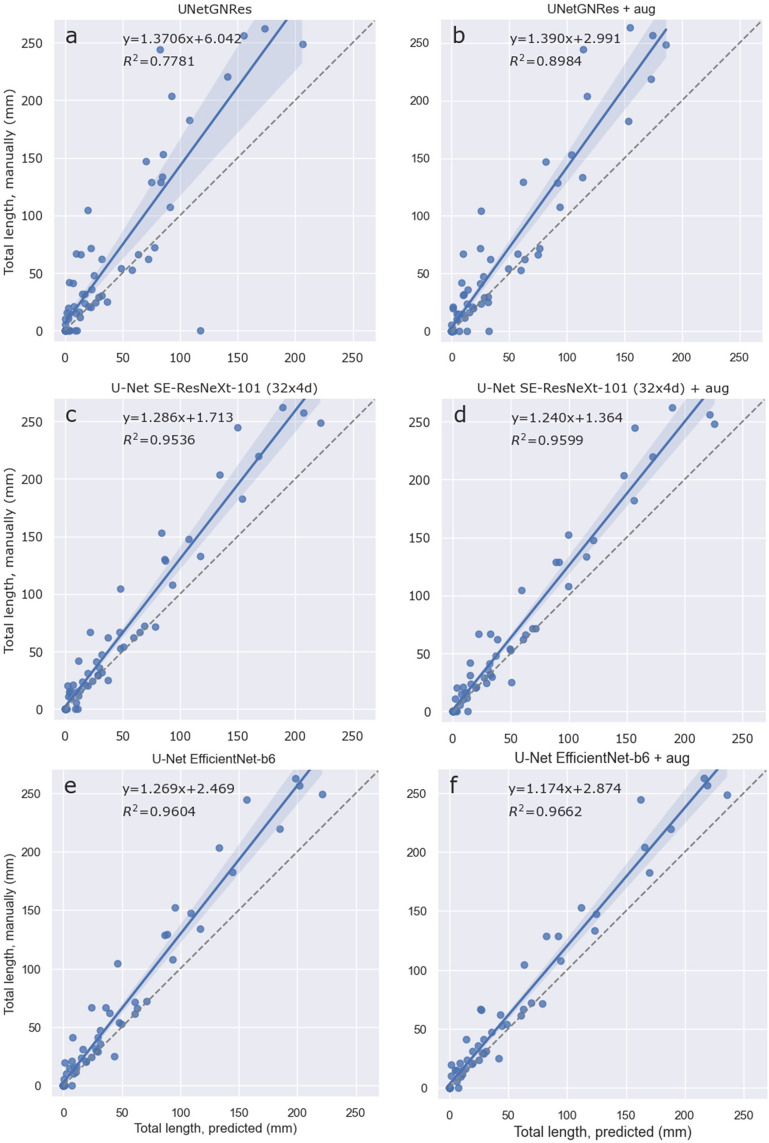
Regression of total root length (mm) per image as derived from manually human labelled masks and as predicted by U-Net models (Table 4) on the mixed test dataset. **a**, **c** and **e** are U-Net models with default (UNetGNRes), SE-ResNeXt-101 and EfficientNet-b6 decoders trained without augmented data, respectively; **b**, **d** and **f** are the corresponding models trained with augmented data (+ aug). Formulas indicate the slope and offset of linear regressions; shaded areas represent 95% confidence interval. Models predict less root length than manually labelled masks. The 1:1 line is shown as a dashed line; R.^2^ values indicate goodness of fit (n = 69)

The dummy classifier used on the mixed dataset shows higher average DSC/IoU scores compared to the *Zea mays* dataset due to the presence of “soil only” images in the mixed set—corresponding to approximately 30% of the total. Frangi Vesselness performed worse than all other methods, making it the least applicable of all methods. In contrast, Adaptive thresholding performed better, but did not reach the minimal accuracy of the dummy classifier when applied to the mixed data set. Surprisingly, SegRoot performed similar to the dummy classifier. While the performance of SegRoot on the mixed dataset was thus limited, an R^2^ of nearly 0.98 was previously reported for a root length determination task on 42 more homogeneous images [38]. Similarly, the SVM performed worse compared to the higher index values obtained on the *Zea mays* dataset. For the SVM algorithm, SegRoot and adaptive thresholding, this indicates that they are less applicable to more diverse MR datasets while performing better on homogeneous data (Tables 1, 2). In contrast, the UNetGNRes model of the well-established RootPainter tool [20] performed well and improved further when augmented data were added. However, the backboned U-Net models had the highest performance metrics when trained on original data and improved further when trained with augmented data (Table 2, Figs. 3, 4).

Both models with non-default encoders show very similar metrics, with the EfficientNet-b6 encoder performing slightly better than the SE-ResNeXt-101 (32 × 4d) encoder. Root length predictions thus reach R^2^ of 0.95–96 in the backboned U-Net models. The UNetGNRes model achieves a good R^2^ of 0.89 when trained with augmented data (Fig. 4). This is similar to [33], where an R^2^ of 0.9217 was achieved for approximate root lengths (grid counts) when trained on 29 images.

### Application of models to new data

Ultimately, the general applicability and robustness of ML approaches can be assessed by applying models trained on one dataset are applied to another. Here, we tested the models trained on the mixed dataset on unseen and distinct *Cichorium intybus* (RootPainter) data (Fig. 2, Table 3). Our results show in particular a robust performance of the complex U-Net encoders (Table 3)—achieving only slightly lower evaluation scores compared to the mixed data (Table 2) and outperforming the dummy classifier. While the good performance is generally confirmed by the ROC curves, they also show that all techniques have greater difficulty in predicting on unseen *Cichorium* data (Fig. 3c), compared to predicting on the test subset of the mixed data (Fig. 3b). The dummy classifier appearing in the diagonal indicates that true negatives are predicted, i.e., those corresponding to soil. In general, the best performance was again observed for the models trained on the augmented dataset, suggesting that the generalisation potential and robustness of a model is substantially improved by additional training data. However, pre-training the models with ImageNet may have contributed substantially. Pre-trained SegRoot was unable to cope with the diverse dataset, whereas UNetGNRes, which did not include pre-training, was able to keep good performance. This suggests that differences in the model architectures, rather than pre-training, played a major role in fitting the data.

**Table 3:** The performance of different techniques/models (Table 4) trained on mixed data (incl. different species, soil types and imaging devices), predicting a different, unseen Chicory/RootPainter dataset

Technique/Model (+ aug)	SSIM	DSC	IoU	FPR
Dummy classifier^b^	0.9732	0.4103	0.4103	–
Frangi Vesselness	0.3361	0.0054	0.0042	0.9984
Adaptive thresholding	0.9408	0.1506	0.1199	0.8814
SVM	0.8260	0.0845	0.0775	0.8862
SegRoot^b^	0.9732	0.4103	0.4103	–
SegRoot + aug^a,b^	0.9732	0.4103	0.4103	–
UNetGNRes	0.9611	0.2489	0.2276	0.5769
UNetGNRes + aug	0.9623	0.3707	0.3022	0.6858
U-Net SE-ResNeXt-101 (32 × 4d)	0.9764	0.4750	0.4156	0.3125
U-Net SE-ResNeXt-101 (32 × 4d) + aug	0.9780	0.5676	0.5043	0.1442
U-Net EfficientNet-b6	0.9771	0.5350	0.4739	0.1827
U-Net EfficientNet-b6 + aug	**0.9784**	**0.6103**	**0.5411**	**0.1233**

^a^No differences in model performance were found using + aug in the SegRoot model

^b^Excluded from FPR determination as not predicting roots. Indicates methods tghat do not predict roots. The best scores for the evaluation metrics average structural similarity index (SSIM), average Sørensen-Dice similarity coefficient (DSC), and average Jaccard index/intersection over union (IoU), and the lowest false positive rate (FPR) are shown in bold. Number of images: Training: 557/augmented Training: 2228, Validation: 62, Testing: 1537. Models trained with augmented data are indicated by + aug

**Fig. 5:**
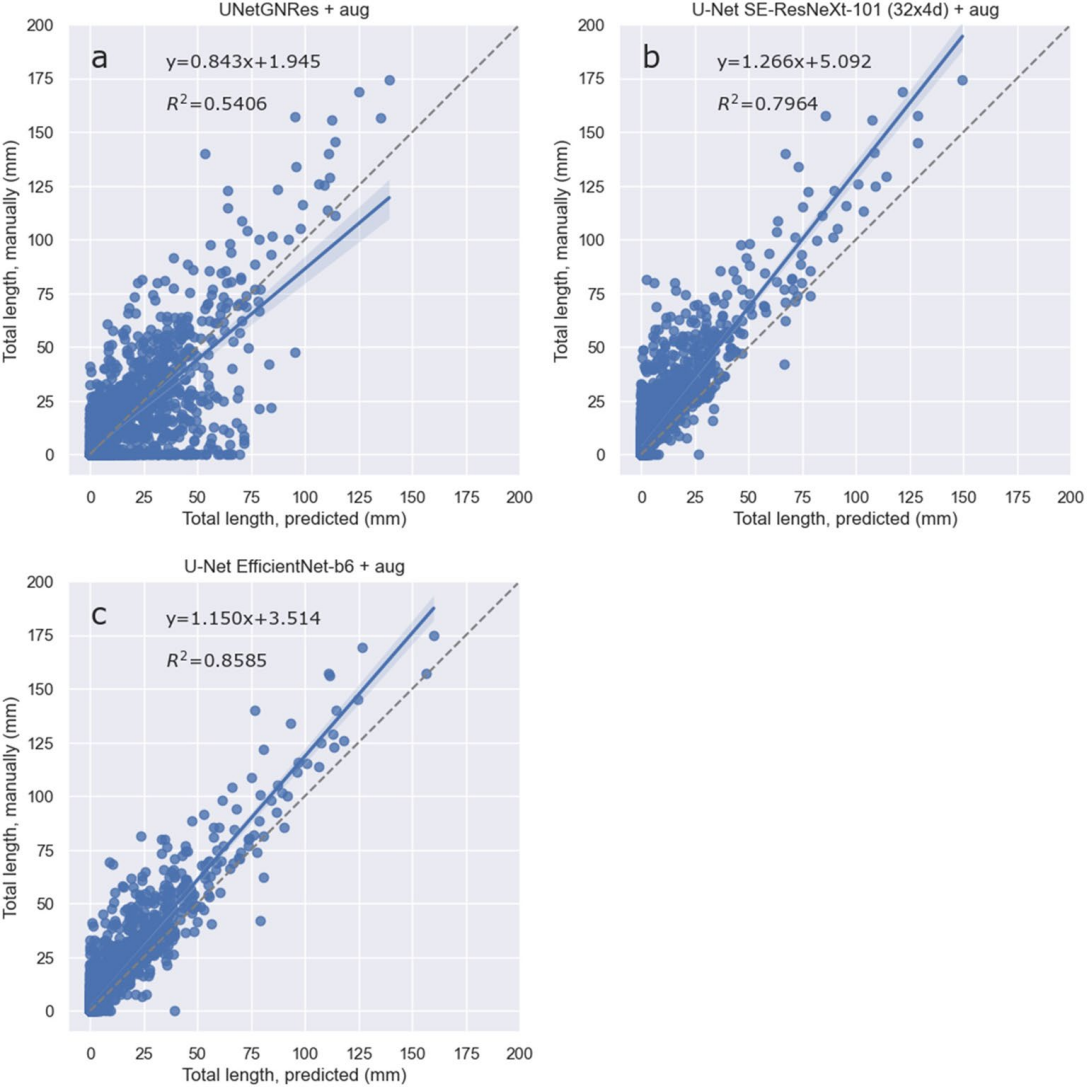
Regression of total root length (mm) per image as derived from manually, human labelled masks and as predicted by the best models, all trained on augmented mixed data (Table 2), predicting on unseen, different *Cichorium intybus* (RootPainter) dataset **a**, **b** and **c** are U-Net models with default (UNetGNRes), SE-ResNeXt-101 and EfficientNet-b6 decoders trained on augmented data, respectively. Formulas indicate the slope and offset of linear regressions; shaded areas represent confidence interval at 95%. Models predict less root length than manually labelled masks. The 1:1 line is shown as a dashed line. R^2^ values indicate goodness of fit (n = 1537). See Table 3 for evaluation metrics

Regarding the root length prediction on unseen data, the best models trained with augmented data are shown in Fig. 5. All models are, however, predict overall fewer roots compared to the ground truth. Thus, all tested methods require manually labelled masks to determine correction factors. However, converting the segmentation output into an accurate root length estimate by skeletonisation can also lead to different length estimates depending on the approach and root orientation [50], but this was not the focus of this study. In addition, the performance of models and the evaluation of any segmentation technique depends on annotation quality [51]. Due to the complexity of root systems and the partial coverage of roots at the root-soil interface, even experienced annotators may introduce errors. Although we manually checked all images for severe masking errors prior to applying the methods, more correct annotations are likely to further improve the accuracy of the segmentation.

### False positives

An important aspect of accurate segmentation is false positives at the image level. Even if only a few pixels of a root-free soil area are incorrectly segmented as a root, this has severe consequences when analysing MR images for root system architecture. While reliably identifying the locations of root tips / end points remains a bottleneck in automated root system analysis of larger rhizotron (or rhizobox) images (which often contain complete root systems) [52], the approximate to these “end points” of a root system on often considerable smaller MR images is the presence or absence of single root segments. Thus, false positives on small MR images will lead to an overestimation of the spatial extent of root systems, e.g. expressed as convex hull [53], and thus to an overestimation of the soil (volume) explored. Our comparison highlighted that there are significant differences between the methods in terms of image-wide false positive rates (Tables 2, 3). While neither the dummy classifier nor SegRoot predicted any roots and were therefore excluded from the FPR analysis, it is striking that Frangi Vesselness, Adaptive thresholding and SVM have very high image-wide FPRs, indicating that some roots were wrongfully predicted in the majority of “soil only” images (Tables 2, 3). Similarly, and surprisingly, the applied UNetGNRes model also had high FPRs ranging from 0.45–0.68, with higher values when predicting on unseen data. This pattern is illustrated in Fig. 5a, showing a range of root lengths predicted when manually annotated images contained no roots. As a workaround, this problem has recently motivated the definition of minimum root length thresholds, e.g. to determine rooting depth [54]. We suggest that the improvements in FPR for the custom U-Net approaches tested are due to the use of pre-trained weights, architectural innovations present in the more modern EfficientNet backbone and the use of a learning rate scheduler, which is likely to help avoid getting stuck in difficult regions of the training process, leading to slow or no improvement in model performance [55]. Learning rate schedulers also allow the use of a larger initial learning rate, which can have a smoothing effect on the model parameters, preventing overfitting and potentially further improving models generalisation [56]. In contrast to UNetGNRes, the U-Net model with EfficientNet-b6 backbone had a very low image-wide FPR of < 0.05 when trained on augmented data (Table 2); error rates increased to ~ 0.12 when the model was applied to unseen data (Table 3, Fig. 5c). Our results emphasise that high FPR is an intrinsic factor of established methods. While image-wide FPR is currently not evaluated in relation to root segmentation tasks, to the best of our knowledge, and potentially because of the rather minor effects on the overall root length prediction, the consequences for the correct spatial mapping of the root system using small MR images are serious and thus deserve further attention.

## Conclusions

To compare the performance of different established techniques for segment roots on (mini-) rhizotron images we assembled three datasets, a standard homogeneous *Zea mays* dataset, a unique mixed MR dataset composed of different soils, plant roots, artefacts, and image qualities, and a dataset of *Cichorium intybus* roots. While previous approaches have often excluded images without roots or containing artefacts from the training and validation datasets [33], this generally limits the applicability of models to real-world MR datasets, which also contain root free soil and artefacts. We therefore suggest following the approach taken here, reducing the number of artefacts and root-free images to a level where these non-root elements are not the dominant image type, but still retaining these standard image situations for model development. While open questions remain about the impact of artefacts on model training, the performance of deep learning models on these more realistic datasets was found to be significantly better than “classic” image processing techniques. The adaptive thresholding technique performed well on the homogeneous Maize dataset, while the Frangi Vesselness filter was not found to be useful for root segmentation under the given conditions. The SVM algorithm evaluated was found to offer only marginal benefits over regular adaptive thresholding. The best techniques for root segmentation were artificial neural networks, with the novel models achieving the highest scores for root detection and length calculation. While the default U-Net showed good results, the novel complex and larger backboned U-Net models were more consistent and robust enough to predict on unseen, distinct rhizotron *Cichorium* images. We can only hypothesize that the inclusion of “soil only” images and images with artefacts in the mixed dataset may underlie the partially different performance of the established models, in particular the SegRoot model. All models, however, detected less roots compared to the manually annotated “ground truth”, and therefore require the application of a correction factor. More problematically, certain techniques exhibit high rates of false positives, which can impede the accurate spatial characterisation of root system architecture on small MR images. Finally, it is important to consider the hardware limitations of our study, as the accordingly limited image sizes are likely restricting the performance of the DL models [49, 57]. Larger images would contain more pixel information and more context for the DL models, which could lead to better performance, and probably improve the average accuracy of all models compared here. It remains open how the inclusion of distinct root classes within the same image set (e.g., white and dark roots) will affect the performance of different model architectures. The establishment of a standardized benchmark dataset of annotated (mini-)rhizotron images is key to facilitate the development of generalisable root image analysis pipelines.

## Methods

### (Mini-)rhizotron datasets: Species, soil types and image properties

Four different (mini-)rhizotron datasets, covering > 8 species (mixtures), 6 soil types, and 4 imaging systems (as detailed below and in Additional file 2), were used in this study.

### ATTRACT project—commercial minirhizotron camera data (Zea mays; *Solanum lycopersicum*, *Vitis vinifera*, *Olea europaea*)

The MR data from Sde Boker Campus, Israel, collected at Ben-Gurion University of the Negev (BGU) facilities, consists of maize (corn; *Zea mays*) in a coarse sandy red-coloured soil (“ATTRACT 1”; Fig. 1), and tomato (*Solanum lycopersicum*) in a coarse sandy yellow soil, grapevine (*Vitis vinifera*) and olive (*Olea europaea*) in a beige loess soil (“ATTRACT 2”). A manual MR camera was used to capture high-resolution images of 2340 × 2400 pixels (UHD), with a very close focus of 23 × 23 mm (VSI MS-190; Vienna Scientific Instruments GmbH, Alland, Austria). The images contained limited amounts of artefacts such as water droplets and small (often white) stones. Roots on 222 images were masked; 115 soil images (i.e., images without annotator-detected roots) were also included in the final dataset. In total, 337 images were used, covering three soil colours and textures, holding roots of one of four species or no roots (“Soil only”) were used.

### MANIP project—custom minirhizotron camera data (tree-grass ecosystem)

The Majadas de Tiétar (MANIP experiment) MR dataset was collected from 10 custom-built automated MR prototypes in a semi-arid tree-grass ecosystem in central Spain [58, 59]. The original dataset used here comprises 250 images (2592 × 2944 pixels; Fig. 1) accompanied by masks created by student assistants. Images were collected using a miniature autofocus camera (DFK AFU050-L34; The Imaging Source GmbH, Bremen, Germany) which was modified with a custom fisheye lens to shorten the field of view. The images were collected during a Mediterranean autumn and winter (2019–2020), and show a variety of illumination/contrast patterns as the soil rewets after the summer drought. There is also a large amount of root litter, making it difficult for human annotators to identify roots. After filtering, 25 images were selected and these were cropped into 6 overlapping images of 972 × 972 pixels each to approximate a similar zoom as the ATTRACT data—increasing the image set to 150 images.

### SegRoot—commercial minirhizotron scanner data (Glycine max)

The SegRoot’s MR dataset includes 65 images of 2550 × 2273 pixels and the same number of masks. Images held soybean (*Glycine max*) roots in a mixture of potting soil and calcined clay gravel, grown in a greenhouse (Department of Botany and Plant Pathology, Purdue University, West Lafayette, USA). The large images were taken using a scanner-based rhizotron imaging system (CI-600, CID Bioscience, Camas, WA, USA). The images were cropped into 64 smaller sections each to match the focus of other datasets. The resulting 4160 images were filtered to 200 images to reach a balanced mixed image set.

### RootPainter—rhizotron compact camera data (*Cichorium intybus*)

RootPainter’s rhizobox dataset consists of 48 images and same number of masks of chicory (*Cichorium intybus*) roots; images with a size of 3991 × 1842 pixels were taken in the summer 2016 at a rhizotron/large rhizobox facility at the University of Copenhagen, Taastrup, Denmark [33]. A compact camera (Olympus Tough TG 860) was used for imaging. Due to the different focus and rectangular shape of the images, each image was divided of each image into 32 smaller images, resulting in a data set of 1536 images.

### Data pre-processing, masks, compilation of image sets for testing, validation, and training

Root images from all datasets were accompanied by masks where a pixel identified as a root was marked as 1 and background as 0. The background included pixels that were fine soil, stones or non-soil artefacts such as petioles/branches, worms, plastic debris, scratches, and deep voids. Images that were obviously inaccurately masked were removed from the datasets by manual screening all images for unmasked root segments prior to analysis. In addition, images containing predominantly non-soil artefacts, such as scratches on the acrylic surface or black plastic foil, were reduced in number to achieve a balanced design; the majority of images in the latter category were present in the SegRoot dataset. All images and masks from the different datasets were rescaled to 256 × 256 pixels, normalised between 0 and 1 and stored as arrays. The image size was set according to the (limited) hardware capacity of the NVIDIA GeForce RTX 2080 Max-Q Design used as GPU.

Three image sets were generated for the analyses: (i) an image set consisting only of *Zea mays* data (ATTRACT 1), which contained 165 images, and presents bright, focused images of white roots in one soil type; (ii) a mixed image set, which containing three different MR datasets in roughly balanced amounts (ATTRACT 2, MANIP, SegRoot) and totalling 687 images; and (iii) the RootPainter *Cichorium* data (1536 images; Additional file 2).

The *Zea mays* image set was divided into training and validation subsets (90% and 10%, respectively). The mixed image set, on the other hand, was created to contain a variety of difficulty levels. The majority of the images (~ 50%) were derived from the ATTRACT dataset as it contains high quality images of 2 soil types and three plant species. The smallest proportion (~ 20%) came from the MANIP dataset, which contained lower quality images that were considered more difficult to segment; SegRoot data contributed around 30%. Before applying the prediction methods, the mixed image set was divided into three subsets: training, validation and test data. Recurrent splitting was performed, first between test and training-validation subsets (1:9 ratio), and then splitting the training-validation subset into validation and training subsets (1:9 ratio). To create a larger training set for the four DL models (Table 4), data augmentation was performed on the training subset of the mixed data set. This entailed rotating the images by flipping them horizontally and vertically, and flipping the RGB channel. In addition, random Gaussian blur, vertical movement blur, brightening and darkening were applied to simulate real-world conditions (e.g., unfocused, captured while moving, different illumination levels). Combined, these augmentations multiplied the number of samples in the mixed training subset by 4 (one original plus three augmentations (i.e., horizontally flipping, vertical flipping and RGB channel flipping)). Finally, iii) the *Cichorium intybus* (RootPainter) image set was used as is for testing purposes only (Additional file 2).

**Table 4:** Imaging processing techniques/models tested or developed in this work, source of the methodology and application to root segmentation tasks

Technique/Model	Encoder	Method source	Adapted to root segmentation	Augmented data
Dummy classifier	–	[60]	–	No
Frangi Vesselness	–	[61]	[32]	No
Adaptive thresholding	–	[29]	[28]	No
Support Vector Machine (SVM)	–	[35]	[34]	No
SegRoot	–	[38]	[38]	Yes
UNetGNRes	Default U-Net	[44]	[20, 33]	Yes
U-Net	SE-ResNeXt-101 (32 × 4d)	[62]	*This paper*	Yes
U-Net	EfficientNet-b6	[62]	*This paper*	Yes

Encoders are only present in U-Net models; augmented data has been added for model training only

### Image processing, machine learning and deep learning techniques

As a baseline for comparison, we use a dummy classifier [60] that predicts the most frequent class—in this case, the zero label for the absence of roots (= “soil only “). This simple predictor can reveal the true performance of a method when compared to it, so that the imbalance in labelling that might occur in the data does not interfere with the evaluation of the methods. We then compared two image processing techniques (which require no training), one machine learning technique, and three DL models.

The first image processing technique is Frangi Vesselness [61]—a filtering technique that recognises tubular structures and has previously been evaluated for root segmentation [33]. In this work, the scikit-image implementation of Frangi Vesselness was used on the root images pre-processed with a bilateral filter and the difference between green and blue filters. The second method used is adaptive thresholding, which considers the illumination of parts of the image as opposed to global thresholding [29]. The adaptive thresholding implementation of the OpenCV library [63] was used on greyscale images to directly predict the mask. Thresholds were determined empirically on maize data by selecting the highest dice coefficient on the validation set.

To train the support vector machine (SVM) algorithm, the data were reduced to two dimensions by transforming each image into a matrix consisting of spatial dimensions flattened (i.e. height and width vectorised into one dimension) by colour channels (another dimension), and then reversing the transformation for evaluation. The used SVM algorithm used is from the scikit-learn package [64], and its performance is influenced by the maximum iterance, the regularisation parameter C and the kernel hyperparameters, which were determined empirically, by testing it 100 times and selecting the best performance on validation data.

SegRoot is a deep-learning (DL) model based on SegNet [38], an image segmentation model with VGG16 as encoder and decoder. The pre-trained VGG16, trained on ImageNet data, was used for the weights in the encoding part of the SegRoot-64-5-trans, the model used in this study. The loss function of this model is a 1-Dice coefficient; a learning rate of 0.01, Adam optimizer and a reduce on plateau scheduler was used. Another established DL-model used is UNetGNRes from RootPainter [20, 33]. Its loss function is a combination of the Dice coefficient and the cross entropy function, a learning rate of 0.01, and an SGD optimiser with Nesterov momentum [65] as specified by Smith et al. [33]. However, the RootPainter model was slightly modified by adding a sigmoid activation function at the end. To allow a more consistent comparison with other models, the input image size was reduced from the original 572 × 572 pixels to 324 × 324 pixels, and the output mask was reduced from the original 388 × 388 pixels to 260 × 260 pixels, which is close to the 256 × 256 pixel output used for the other DL models. Thus, the data used for this model was modified by not only padding the input and output already resized arrays, but also by adding an extra array to the mask output. This results in the mask having 2 channels: the mask and its negative image. The padding was removed during evaluaation, so all images were 256 × 256 pixels when evaluated.

Finally, the DL models used in this study are U-Net models implemented in PyTorch with two different encoders as backbones, loaded from the segmentation models’ package [62]. These two U-Net models have considerably more parameters than the previous SegRoot and RootPainter models. The backbones used here are (i) SE-ResNeXt-101 (32 × 4d) and (ii) EfficientNet-b6. These encoders were pre-trained on ImageNet data [62]. The loss function used to train this model is the structural similarity index (SSIM) as suggested by He et al. [66] for road extraction task. The learning rate was 0.0001, Adam [67] was used as optimiser, and Cosine Annealing learning rate as a scheduler [68]. Characteristics of the architectures of the four DL models are summarised in Additional file 3.

All four DL models are trained on the augmented data as described above. All DL models were implemented in the PyTorch library. They were trained for 100 epochs (Additional file 5), and the best model was selected based on the performance obtained on the validation set; this performance is the combination of the structural similarity index (SSIM) and the Jaccard index evaluation metrics (see below). An overview of all methods is given in Table 4.

### Evaluation of the performance

Receiver operating characteristic (ROC) curves and three indices were used to evaluate image segmentation: Sørensen-Dice similarity coefficient (Dice or DSC), Jaccard index or intersection over union (IoU) and structural similarity index (SSIM). The DSC is a simple and useful summary measure of the spatial overlap between two segmentations, A and B target regions, that can measure the accuracy of an image segmentation task [69]; it is defined as

1 $$DSC\left(A,B\right)=\frac{2\left(A\cap B\right)}{A+B}$$

where $\cap$ is the intersection [70]. Using the same terminology as DSC, IoU can be defined as

2 $$\mathrm{J}\left(\mathrm{A},\mathrm{B}\right)=\frac{\left|\mathrm{A}\cap \mathrm{B}\right|}{\left|\mathrm{A}\cup \mathrm{B}\right|}$$

where $\cap$ is the intersection and $\cup$ is the union. The SSIM index calculates the similarity between two images in terms of three features: luminance, contrast and image structure [71]. SSIM is defined as

3 $$S\left(x,y\right)=f\left(l\left(x,y\right),c\left(x,y\right),s(x,y)\right)$$

where l, c and s correspond to luminance, contrast, and structure respectively. All three metrics have values between 0 and 1, higher values indicating better performance. Averages of DSC, IoU and SSIM are given.

ROC curves are another way to evaluate the performance of the predictions made by different techniques. We used ROC to visualise the true positive rate vs. the false positive rate of each technique`s predictions [72]. These plots show not only how good the prediction is (the closer to the (0,1) position in the top left corner, the better), but also how “conservative” (i.e. classifying as positive only with strong evidence) or “liberal” (i.e. classifying as positive even with weak evidence) each method is [72]. ROC curves are insensitive to class imbalance [72], which is a characteristic of our data set as most of the pixels are background and very few pixels are roots.

False positive rate (FPR) was defined as

4 $$FPR=\frac{FP}{FP+TN}$$

where FP are false positives, and TN are true negatives. In this study, this metric will reflect the error percentage of “soil only” images falsely classified as containing roots.

Finally, the predicted total root length was evaluated by skeletonising the masks using the scikit-image library [73] and the posterior sum of the pixels and multiplied by the pixel-mm relation, with each MR dataset having a different one (data not shown). Total root length was calculated on the mixed test set and on the unseen *Cichorium intybus* test set. The predictions of the best methods were compared with the original segmentation based on human-labelled roots, and R^2^ was calculated from the regression between the two variables.

### Supplementary Information

**Additional file 1: **Systems used to capture images within minirhizotron tubes, manufacturers and models, resolution, image size and spectral range. Table.

**Additional file 2: **Use of (mini-) rhizotron (MR) image datasets, species present and number of soil types, and their grouping into three image sets, for training, validation and/or testing. Table.

**Additional file 3: **Artificial Neural Networks architectures characteristics. Table.

**Additional file 4: **Masks prediction examples on validation or test data of the image dataset with (4.1–4.4) or without (4.5) roots. 5 images.

**Additional file 5: **Models training plots for Zea mays (5.1) and mixed data (5.2). 2 images.

## Data Availability

The ATTRACT minirhizotron dataset generated during the current study is available from PB; other minirhizotron datasets are available from original sources. The dataset supporting the conclusions of this article is available from PB on reasonable request.

## References

[1] Smit AL, Bengough AG, Engels C, van Noordwijk M, Pellerin S, van de Geijn SC (2013). Root methods: a handbook.

[2] Ryan PR, Delhaize E, Watt M, Richardson AE (2016). Plant roots: understanding structure and function in an ocean of complexity. Ann Bot.

[3] Freschet G, Roumet C, Comas L, Weemstra M, Bengough A, Rewald B (2021). Root traits as drivers of plant and ecosystem functioning: current understanding, pitfalls and future research needs. New Phytol.

[4] Schroth G (1998). A review of belowground interactions in agroforestry, focussing on mechanisms and management options. Agrofor Syst.

[5] Wijesinghe DK, John EA, Hutchings MJ (2005). Does pattern of soil resource heterogeneity determine plant community structure?. An experimental investigation J Ecol.

[6] McCormack ML, Dickie IA, Eissenstat DM, Fahey TJ, Fernandez CW, Guo D (2015). Redefining fine roots improves understanding of below-ground contributions to terrestrial biosphere processes. New Phytol.

[7] Pierret A, Doussan C, Capowiez Y, Bastardie F, Pagès L (2007). Root functional architecture: a framework for modeling the interplay between roots and soil. Vadose Zone J.

[8] Lynch JP, Strock CF, Schneider HM, Sidhu JS, Ajmera I, Galindo-Castañeda T (2021). Root anatomy and soil resource capture. Plant Soil.

[9] Atkinson JA, Pound MP, Bennett MJ, Wells DM (2019). Uncovering the hidden half of plants using new advances in root phenotyping. Curr Opin Biotechnol.

[10] Mooney SJ, Pridmore TP, Helliwell J, Bennett MJ (2012). Developing X-ray computed tomography to non-invasively image 3-D root systems architecture in soil. Plant Soil.

[11] Amato M, Basso B, Celano G, Bitella G, Morelli G, Rossi R (2008). *In situ* detection of tree root distribution and biomass by multi-electrode resistivity imaging. Tree Physiol.

[12] Freschet GT, Pagès L, Iversen CM, Comas LH, Rewald B, Roumet C (2021). A starting guide to root ecology: strengthening ecological concepts and standardising root classification, sampling, processing and trait measurements. New Phytol.

[13] Withington JM, Elkin AD, Bułaj B, Olesiński J, Tracy KN, Bouma TJ (2003). The impact of material used for minirhizotron tubes for root research. New Phytol.

[14] Svane SF, Dam EB, Carstensen JM, Thorup-Kristensen K (2019). A multispectral camera system for automated minirhizotron image analysis. Plant Soil.

[15] Rahman G, Sohag H, Chowdhury R, Wahid KA, Dinh A, Arcand M (2020). SoilCam: a fully automated minirhizotron using multispectral imaging for root activity monitoring. Sensors.

[16] Arnaud M, Baird AJ, Morris PJ, Harris A, Huck JJ (2019). EnRoot: a narrow-diameter, inexpensive and partially 3D-printable minirhizotron for imaging fine root production. Plant Methods.

[17] Sell M, Smith AG, Burdun I, Rohula-Okunev G, Kupper P, Ostonen I (2022). Assessing the fine root growth dynamics of Norway spruce manipulated by air humidity and soil nitrogen with deep learning segmentation of smartphone images. Plant Soil..

[18] Zeng G, Birchfield ST, Wells CE (2008). Automatic discrimination of fine roots in minirhizotron images. New Phytol.

[19] Möller B, Chen H, Schmidt T, Zieschank A, Patzak R, Türke M (2019). rhizoTrak: a flexible open source Fiji plugin for user-friendly manual annotation of time-series images from minirhizotrons. Plant Soil.

[20] Smith AG, Han E, Petersen J, Olsen NAF, Giese C, Athmann M (2022). RootPainter: deep learning segmentation of biological images with corrective annotation. New Phytol.

[21] Le Bot J, Serra V, Fabre J, Draye X, Adamowicz S, Pagès L (2010). DART: a software to analyse root system architecture and development from captured images. Plant Soil.

[22] Lobet G, Pagès L, Draye X (2011). A novel image-analysis toolbox enabling quantitative analysis of root system architecture. Plant Physiol.

[23] Armengaud P (2009). EZ-Rhizo software: the gateway to root architecture analysis. Plant Signal Behav.

[24] Bucksch A, Burridge J, York LM, Das A, Nord E, Weitz JS (2014). Image-based high-throughput field phenotyping of crop roots. Plant Physiol.

[25] Galkovskyi T, Mileyko Y, Bucksch A, Moore B, Symonova O, Price CA (2012). GiA Roots: software for the high throughput analysis of plant root system architecture. BMC Plant Biol.

[26] Pound M, French A, Atkinson J, Wells D, Bennett M, Pridmore T (2013). RootNav: navigating images of complex root architectures. Plant Physiol.

[27] Borianne P, Subsol G, Fallavier F, Dardou A, Audebert A (2018). GT-RootS: An integrated software for automated root system measurement from high-throughput phenotyping platform images. Comput Electron Agric.

[28] Narisetti N, Henke M, Seiler C, Shi R, Junker A, Altmann T (2019). Semi-automated root image analysis (saRIA). Sci Rep.

[29] Bradley D, Roth G (2007). Adaptive thresholding using the integral image. Journal of graphics tools.

[30] Frangi R, Niessen WJ, Vincken K, Viergever M (2000). Multiscale vessel enhancement filtering. Med Image Comput Comput Assist Interv.

[31] Chapman BE, Parker D. An analysis of vessel enhancement filters based on the hessian matrix for intracranial MRA. Proc Soc Magn Reson Med 2001. p. 607.

[32] Schulz H, Postma JA, van Dusschoten D, Scharr H, Behnke S, Csurka G, Kraus M, Laramee RS, Richard P, Braz J (2013). Plant root system analysis from MRI images. Computer vision, imaging and computer graphics theory and application.

[33] Smith A, Petersen J, Selvan R, Rasmussen C (2020). Segmentation of roots in soil with U-Net. Plant Methods.

[34] Yu G, Zare A, Sheng H, Matamala R, Reyes-Cabrera J, Fritschi FB (2020). Root identification in minirhizotron imagery with multiple instance learning. Mach Vis Appl.

[35] Hearst MA, Dumais ST, Osuna E, Platt J, Scholkopf B (1998). Support vector machines. IEEE Intell Syst.

[36] Dargan S, Kumar M, Ayyagari MR, Kumar G (2020). A survey of deep learning and its applications: a new paradigm to machine learning. Arch Comput Methods Eng.

[37] Shen C, Liu L, Zhu L, Kang J, Wang N, Shao L (2020). High-throughput in situ root image segmentation based on the improved deeplabv3+ method. Front Plant Sci.

[38] Wang T, Rostamza M, Song Z, Wang L, McNickle G, Iyer-Pascuzzi AS (2019). SegRoot: a high throughput segmentation method for root image analysis. Comput Electron Agric.

[39] Ward D, Moghadam P (2020). Scalable learning for bridging the species gap in image-based plant phenotyping. Comput Vis Image Underst.

[40] Tan M, Le Q. Efficientnet: Rethinking model scaling for convolutional neural networks. Proceedings of the 36th International Conference on Machine Learning. 97. Long Beach: PMLR; 2019. p. 6105–14.

[41] Hu J, Shen L, Sun G, editors. Squeeze-and-Excitation Networks. 2018 IEEE/CVF Conference on Computer Vision and Pattern Recognition PP: 7132–7141 doi: 101109/CVPR201800745; 2018 18–23 June 2018.

[42] Bianco S, Cadene R, Celona L, Napoletano P (2018). Benchmark analysis of representative deep neural network architectures. IEEE Access.

[43] Huynh LD, Boutry N. A U-Net++ With Pre-Trained EfficientNet Backbone for Segmentation of Diseases and Artifacts in Endoscopy Images and Videos. EndoCV@ISBI. Iowa City, Iowa, USA.

[44] Ronneberger O, Fischer P, Brox T, Navab N, Hornegger J, Wells WM, Frangi AF (2015). U-Net: convolutional networks for biomedical image segmentation. Medical Image computing and computer-assisted intervention—MICCAI 2015.

[45] Wang J, Zhang X, Lv P, Zhou L, Wang H. EAR-U-Net: EfficientNet and attention-based residual U-Net for automatic liver segmentation in CT. ArXiv. 2021. abs/2110.01014.

[46] Baheti B, Innani S, Gajre S, Talbar S. Eff-UNet: A Novel architecture for semantic segmentation in unstructured environment. IEEE/CVF Conference on Computer Vision and Pattern Recognition Workshops (CVPRW) 2020. p. 1473–81.

[47] Shorten C, Khoshgoftaar TM (2019). A survey on image data augmentation for deep learning. J Big Data.

[48] Badrinarayanan V, Handa A, Cipolla R. Segnet: A deep convolutional encoder-decoder architecture for robust semantic pixel-wise labelling. arXiv preprint 2015: arXiv:1505.07293. 10.1109/TPAMI.2016.2644615 28060704

[49] Luke J, Joseph R, Balaji M (2019). Impact of image size on accuracy and generalization of convolutional neural networks. IJRAR.

[50] Kimura K, Kikuchi S, Yamasaki S-i (1999). Accurate root length measurement by image analysis. Plant Soil..

[51] Tajbakhsh N, Jeyaseelan L, Li Q, Chiang JN, Wu Z, Ding X (2020). Embracing imperfect datasets: a review of deep learning solutions for medical image segmentation. Med Image Anal.

[52] Pound MP, Atkinson JA, Townsend AJ, Wilson MH, Griffiths M, Jackson AS (2017). Deep machine learning provides state-of-the-art performance in image-based plant phenotyping. GigaScience.

[53] Rangarajan H, Lynch JP (2021). A comparative analysis of quantitative metrics of root architecture. Plant Phenomics.

[54] Wacker T, Popovic O, Olsen N, Markussen B, Smith A, Svane S (2021). Semi-field root phenotyping: root traits for deep nitrate uptake. Plant Cell Env.

[55] Smith L. 2017. Cyclical Learning Rates for Training Neural Networks IEEE Winter on Applications of Computer Vision (WACV) Santa Rosa IEEE winter applications of computer vision. 10.1109/WACV.2017.58.

[56] Smith L, Topin N. Super-convergence: very fast training of neural networks using large learning rates: SPIE; 2019.

[57] Hamwood J, Alonso-Caneiro D, Read SA, Vincent SJ, Collins MJ (2018). Effect of patch size and network architecture on a convolutional neural network approach for automatic segmentation of OCT retinal layers. Biomed Opt Express.

[58] El-Madany TS, Reichstein M, Perez-Priego O, Carrara A, Moreno G, Pilar Martín M (2018). Drivers of spatio-temporal variability of carbon dioxide and energy fluxes in a Mediterranean savanna ecosystem. Agric For Meteorol.

[59] Nair R, Strube M, Hertel M, Kolle O, Rolo V, Migliavacca M (2023). High frequency root dynamics: sampling and interpretation using replicated robotic minirhizotrons. Journal of Experimental Botany.

[60] Audebert N, Le Saux B, Lefèvre S (2017). Segment-before-detect: vehicle detection and classification through semantic segmentation of aerial images. Remote Sens.

[61] Frangi A, Niessen WJ, Vincken K, Viergever M, Wells WM, Colchester A, Delp S (1998). Multiscale Vessel Enhancement Filtering. International on medical image computing and computer-assisted intervention.

[62] Yakubovskiy P. Segmentation models pytorch 2020. https://github.com/qubvel/segmentation_models.pytorch. Accessed 6 July 2022.

[63] The BG, Library OpenCV (2000). The openCV Library. Dr Dobb's J Software Tools.

[64] Pedregosa F, Varoquaux G, Gramfort A, Michel V, Thirion B, Grisel O (2011). Scikit-learn: machine learning in python. J Mach Learn Res.

[65] Ruder S. An overview of gradient descent optimization algorithms. arXiv preprint 2016: arXiv:1609.04747.

[66] He H, Yang D, Wang S, Wang S, Li Y (2019). Road extraction by using atrous spatial pyramid pooling integrated encoder-decoder network and structural similarity loss. Remote Sens.

[67] Kingma DP, Ba J. Adam: A method for stochastic optimization. arXiv preprint 2014: arXiv:1412.6980.

[68] Loshchilov I, Hutter F. Sgdr: Stochastic gradient descent with warm restarts. arXiv preprint 2016:arXiv:1608.03983.

[69] Shamir RR, Duchin Y, Kim J, Sapiro G, Harel N. Continuous dice coefficient: a method for evaluating probabilistic segmentations. arXiv preprint 2019.

[70] Zou KH, Warfield SK, Bharatha A, Tempany CM, Kaus MR, Haker SJ (2004). Statistical validation of image segmentation quality based on a spatial overlap index^1^: scientific reports. Acad Radiol.

[71] Wang Z, Bovik AC, Sheikh HR, Simoncelli EP (2004). Image quality assessment: from error visibility to structural similarity. IEEE Trans Image Process.

[72] Fawcett T (2006). Introduction to ROC analysis. Pattern Recognit Lett.

[73] Van der Walt S, Schönberger J, Nunez-Iglesias J, Boulogne F, Warner J, Yager N (2014). scikit-image: image processing in python. PeerJ.

