# Supplementary material for: Semantic segmentation of plant roots from RGB (mini-) rhizotron images—generalisation potential and false positives of established methods and advanced deep-learning models
Source: Plant Methods. 2023 Nov 6;19:122. doi: 10.1186/s13007-023-01101-2 (PMC10629126; doi:10.1186/s13007-023-01101-2)
Supplement: Supplementary file 1 — Additional file 1: Systems used to capture images within minirhizotron tubes, manufacturers and models, resolution, image size and spectral range. Table. [file 13007_2023_1101_MOESM1_ESM.pdf]

Additional file 1. Systems used to capture images within minirhizotron tubes, manufacturer and models, resolution, image size and spectral range; this list is not exhaustive. References of previous use are exemplified if available

Manufacturer	model	Max. resolution	Image size	Reference
Bartz, LLC (closed)	BTC-2		13 x 18 mm	Hill et al. (2013)
CID Bioscience	CI-600	2550 x 2273 pixels	~20 x 19 cm	Postic et al. (2019)
Rhizosystems LLC	Manual MR	640 x 480 pixels	8.4 x 6.3 mm	Phillips et al. (2019)
Videometer	VideometerMR	5 Mpx	n.a.	Svane et al. (2019)
Vienna Scientific Instruments GmbH	MS-190	2340 x 2400 pixels	23 x 23 mm	Bauer et al. (2022)

n.a., not available

References

Bauer F, Lärm L, Morandage S, Lobet G, Vanderborght J, Vereecken H, Schnepf A (2022) Development and Validation of a Deep Learning Based Automated Minirhizotron Image Analysis Pipeline. Plant Phenomics, 1-14. doi: 10.34133/2022/9758532.

Hill A, Rewald B, Rachmilevitch S (2013) Belowground dynamics in two olive varieties as affected by saline irrigation. Scientia Horticulturae 162: 313-319. doi: 10.1016/j.scienta.2013.08.032.

Phillips ML, McNellis BE, Allen MF, & Allen EB (2019). Differences in root phenology and water depletion by an invasive grass explains persistence in a Mediterranean ecosystem. American Journal of Botany, 106(9), 1210-1218.

Postic F, Beauchêne K, Gouache D, Doussan C (2019) Scanner-based minirhizotrons help to highlight relations between deep roots and yield in various wheat cultivars under combined water and nitrogen deficit conditions. Agronomy 9:297.

Svane SF, Dam EB, Carstensen JM, Thorup-Kristensen K (2019) A multispectral camera system for automated minirhizotron image analysis. Plant and Soil 441: 657-672. doi: 10.1007/s11104-019-04132-8.
