# Supplementary material for: Semantic segmentation of plant roots from RGB (mini-) rhizotron images—generalisation potential and false positives of established methods and advanced deep-learning models
Source: Plant Methods. 2023 Nov 6;19:122. doi: 10.1186/s13007-023-01101-2 (PMC10629126; doi:10.1186/s13007-023-01101-2)
Supplement: Supplementary file 2 — Additional file 2: Use of (mini-) rhizotron (MR) image datasets, species present and number of soil types, and their grouping into three image sets, for training, validation and/or testing. Table. [file 13007_2023_1101_MOESM2_ESM.pdf]

Additional file 2. Use of (mini-)rhizotron (MR) image datasets, present species and number of soil types, and their grouping in three image sets, for training, validation and/or testing

**MR Dataset**	**Species**	**# Soil types**	**Image set**	**Training**	**Validation**	**Testing**
ATTRACT 1	*Zea mays*	1	1 - Corn	**X**	**X**	
ATTRACT 2	*Olea europaea, Solanum lycopersicum, Vitis vinifera*	2	2 -Mixed	**X**	**X**	**X**
MANIP	tree-grassland (div. species)	1	2 - Mixed	**X**	**X**	**X**
SegRoot	*Glycine max*	1	2 - Mixed	**X**	**X**	**X**
RootPainter	*Cichorium intybus*	1	3 - Chicory			**X**
