# Supplementary material for: Semantic segmentation of plant roots from RGB (mini-) rhizotron images—generalisation potential and false positives of established methods and advanced deep-learning models
Source: Plant Methods. 2023 Nov 6;19:122. doi: 10.1186/s13007-023-01101-2 (PMC10629126; doi:10.1186/s13007-023-01101-2)
Supplement: Supplementary file 3 — Additional file 3: Artificial Neural Networks architectures characteristics. Table. [file 13007_2023_1101_MOESM3_ESM.pdf]

Additional file 3. Artificial Neural Networks architectures characteristics. All hyperparameters including learning rate (Lr) and loss function from original architectures (SegRoot, UNetGNRes) were kept as is. All parameters present are trainable. The estimated size shows the memory needed for the training, in addition to the input size, but disregards the potentially substantial batch size used for training which can substantially increase the memory usage

**Architectures**	**Parameters (Millions)**	**Estimated memory size (MB)**	**Loss function**	**Lr**	**Optimizer**	**Scheduler**	**Pre-trained**
SegRoot	24.8	7566.13	Custom dice loss	0.001	Adam	Reduce on plateau	Custom
UNetGNRes	1.3	1015.21	Custom combined dice & cross entropy	0.0001	SGD	-	-
U-Net SE-ResNeXt-101 (32x4d)	55.9	1244.34	1-SSIM	0.0001	Adam	Cosine Annealing	ImageNet
U-Net EfficientNet-b6	42.5	1588.62	1-SSIM	0.0001	Adam	Cosine Annealing	ImageNet
