# Supplementary material for: Semantic segmentation of plant roots from RGB (mini-) rhizotron images—generalisation potential and false positives of established methods and advanced deep-learning models
Source: Plant Methods. 2023 Nov 6;19:122. doi: 10.1186/s13007-023-01101-2 (PMC10629126; doi:10.1186/s13007-023-01101-2)
Supplement: Supplementary file 4 — Additional file 4: Masks prediction examples on validation or test data of the image dataset with (4.1–4.4) or without (4.5) roots. 5 images. [file 13007_2023_1101_MOESM4_ESM.pdf]

**Additional file 4.**

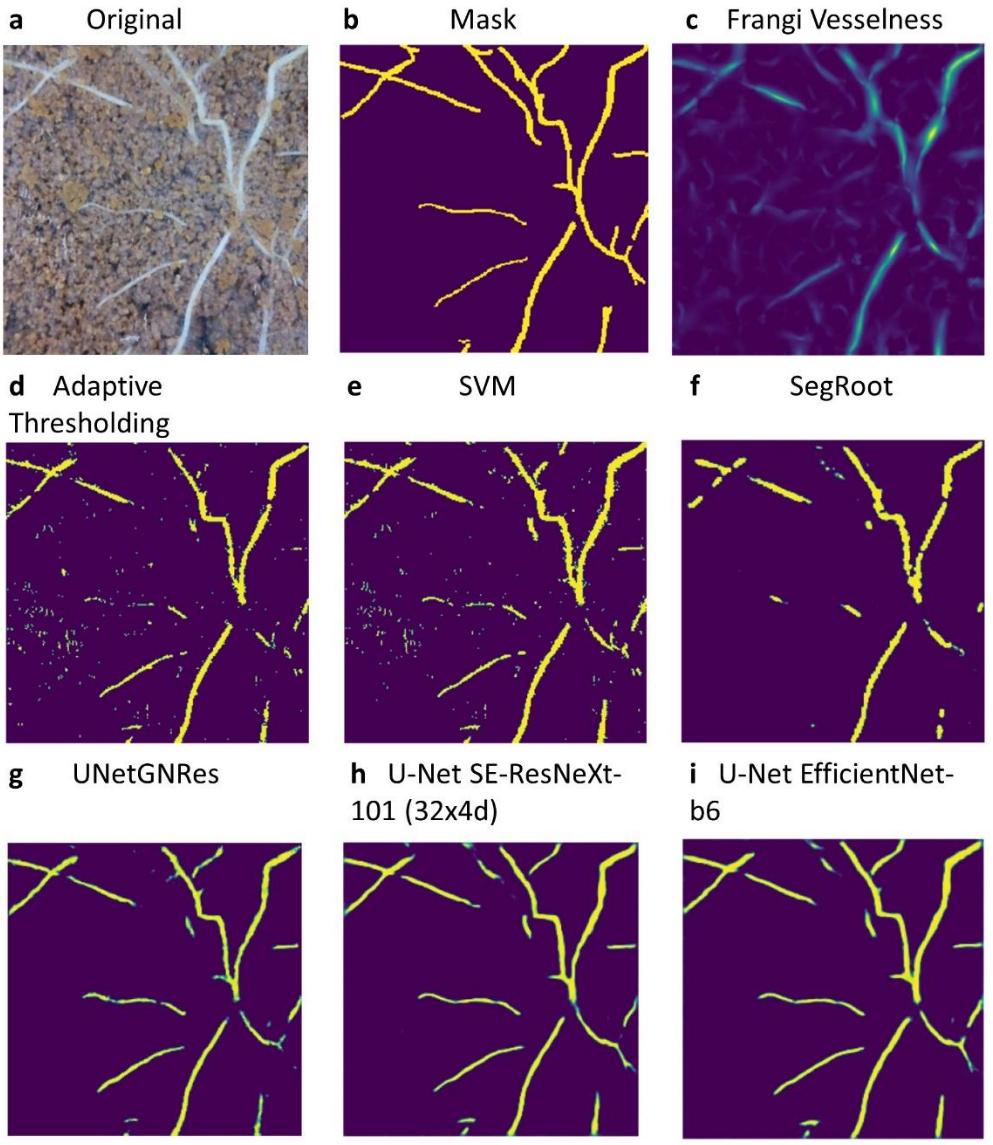


**Additional file 4.1** Masks prediction examples on validation data of the *Zea mays* (ATTRACT 1) dataset. Original image from the MR **a**, manually labelled mask **b**, and masks derived with the techniques/models Frangi Vesselness **c**, Adaptive Thresholding **d**, Support vector machine (SVM) **e**, SegRoot **f**, UNetGNRes **g**, U-Net SE-ResNeXt-101 (32x4d) **h**, and U-Net EfficientNet-b6 **i**; see Table 4 and text for details

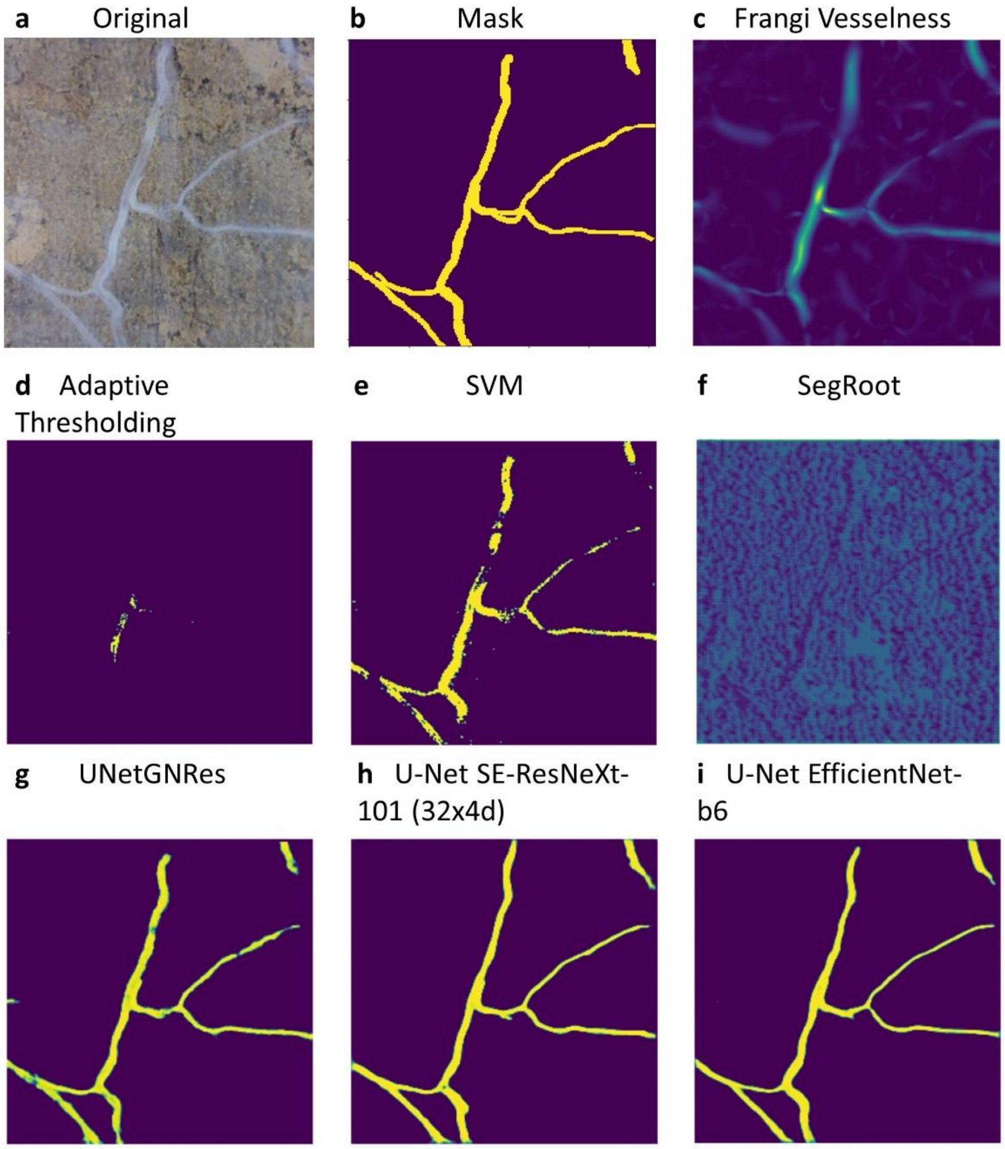


**Additional file 4.2** Masks prediction examples on test data of the ATTRACT 2 dataset (Additional file 2; *Olea europaea, Solanum lycopersicum, Vitis vinifera*). Original image (*Olea europaea*)) from the MR **a**, manually labelled mask **b**, and masks derived with the techniques/models Frangi Vesselness **c**, adaptive thresholding **d**, Support vector machine (SVM) **e**, SegRoot **f**, UNetGNRes **g**, U-Net SE-ResNeXt-101 (32x4d) **h**, and U-Net EfficientNet-b6 **i**; see Table 4 and text for details

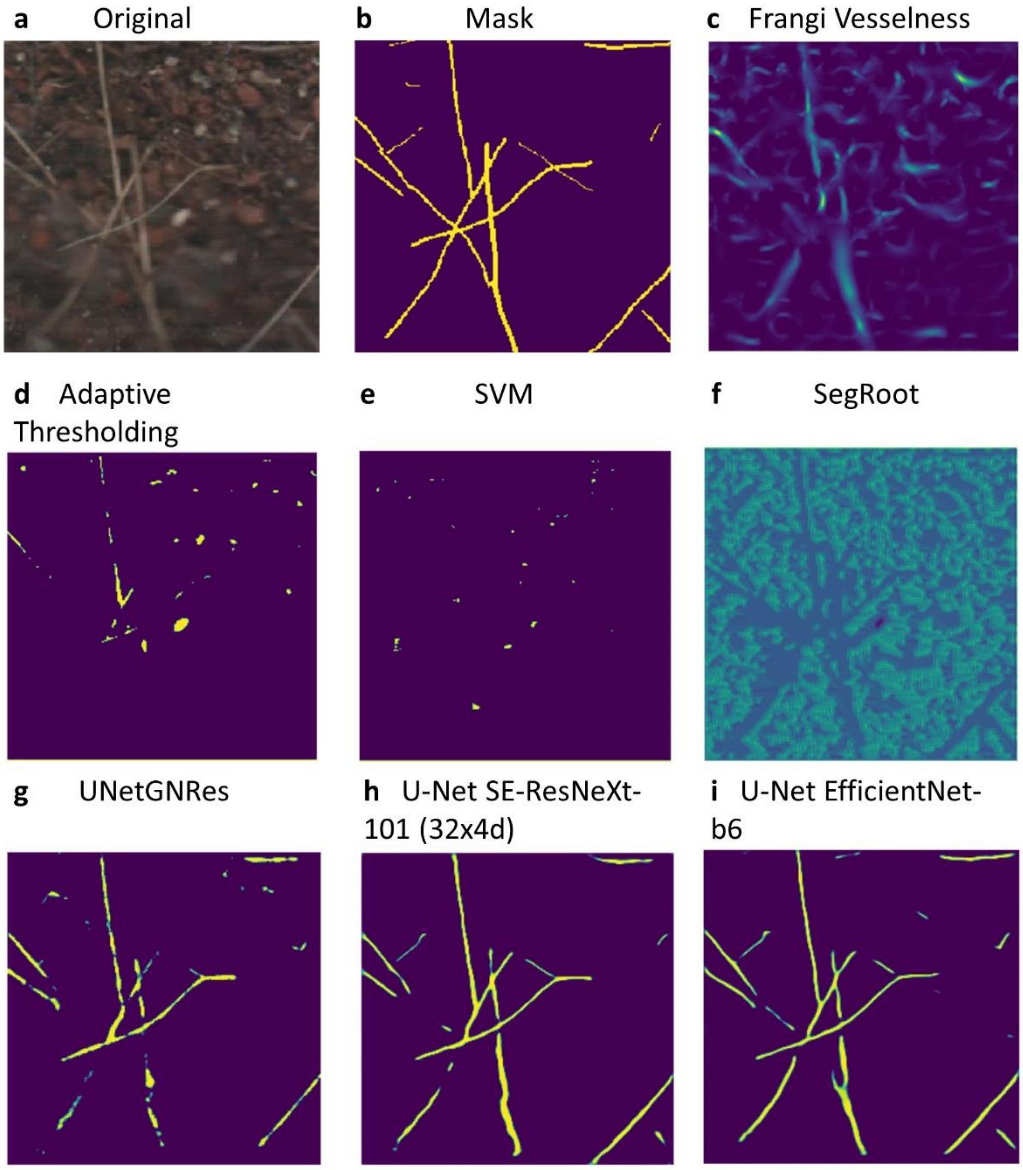


**Additional file 4.3** Masks prediction examples on test data of the Mixed dataset (Additional file 2). Original image (SegRoot, *Glycine max*) from the MR **a**, manually labelled mask **b**, and masks derived with the techniques/models Frangi Vesselness **c**, adaptive thresholding **d**, Support vector machine (SVM) **e**, SegRoot **f**, UNetGNRes **g**, U-Net SE-ResNeXt-101 (32x4d) **h**, and U-Net EfficientNet-b6 **i**; see Table 4 and text for details

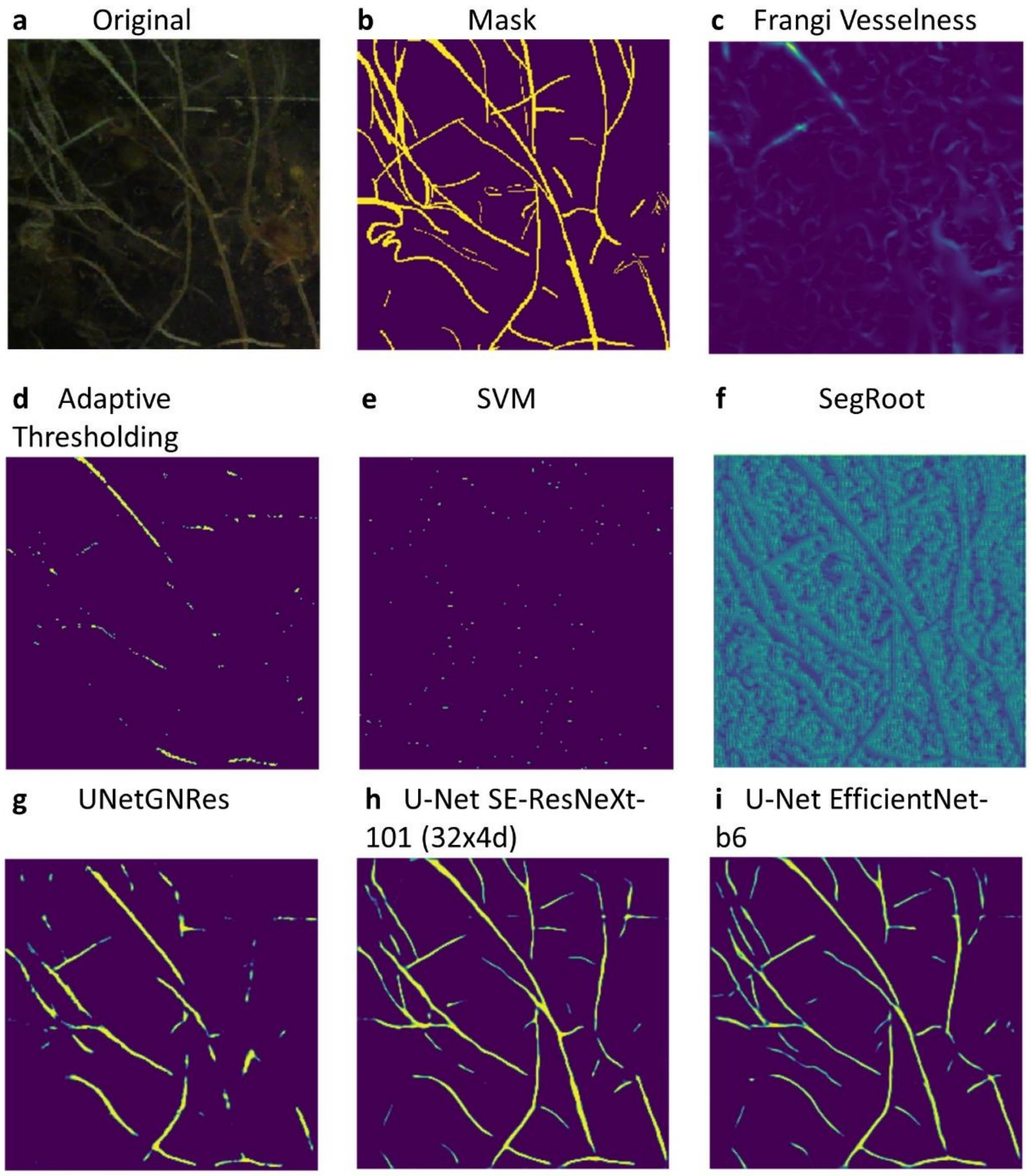


**Additional file 4.4** Masks prediction examples on test data of the Mixed dataset (Additional file 2). Original image (MANIP*, tree-grass ecosystem*) from the MR **a**, manually labelled mask **b**, and masks derived with the techniques/models Frangi Vesselness **c**, adaptive thresholding **d**, Support vector machine (SVM) **e**, SegRoot **f**, UNetGNRes **g**, U-Net SE-ResNeXt-101 (32x4d) **h**, and U-Net EfficientNet-b6 **i**; see Table 4 and text for details

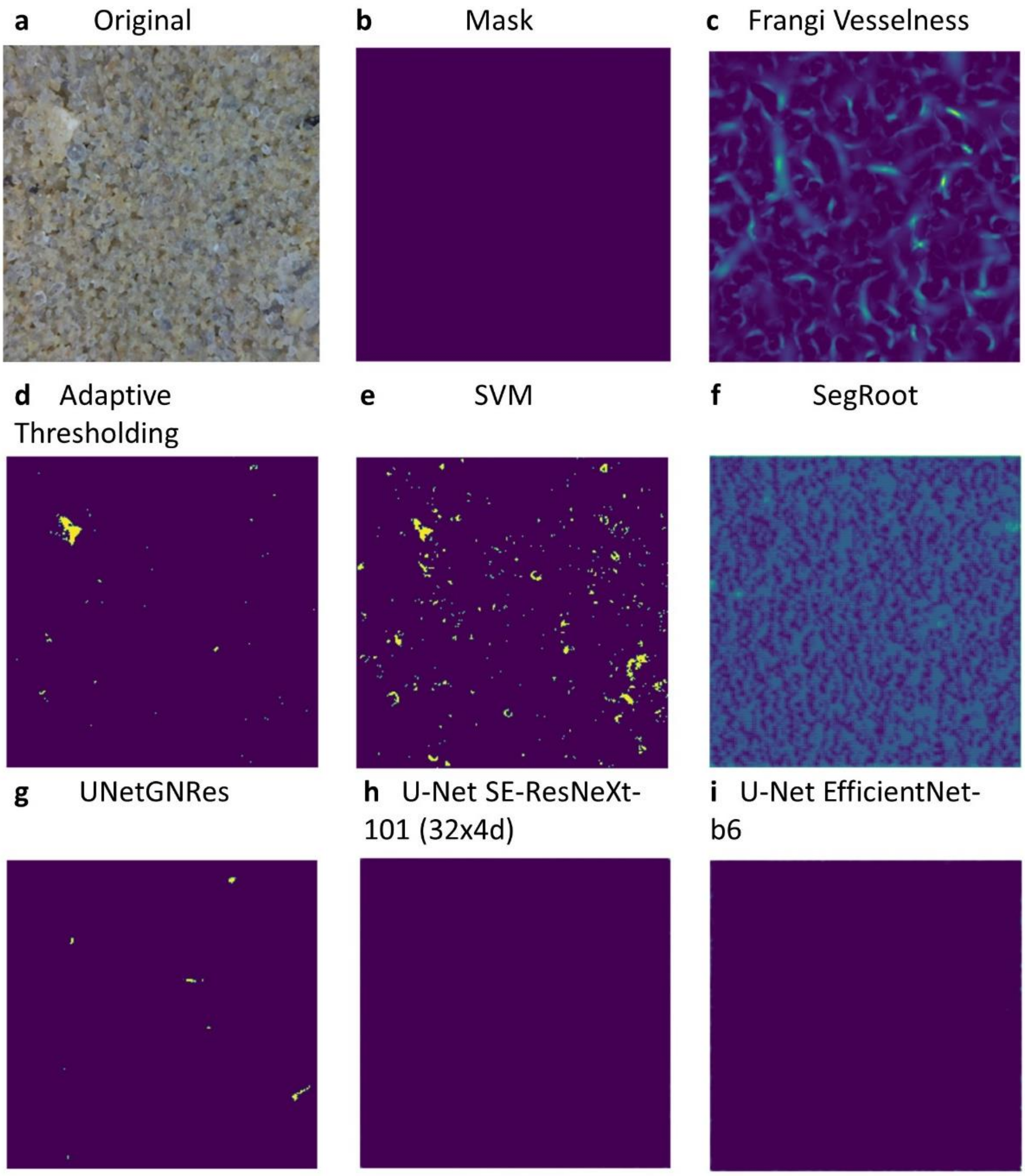


**Additional file 4.5** Masks prediction examples on test data of the ATTRACT 2 dataset without roots (Additional file 2). Original image ("soil-only", with stone artefact) from the MR **a**, manually labelled mask **b**, and masks derived with the techniques/models Frangi Vesselness **c**, adaptive thresholding **d**, Support vector machine (SVM) **e**, SegRoot **f**, UNetGNRes **g**, U-Net SE-ResNeXt-101 (32x4d) **h**, and U-Net EfficientNet-b6 **i**; see Table 4 and text for details
