# Supplementary material for: Semantic segmentation of plant roots from RGB (mini-) rhizotron images—generalisation potential and false positives of established methods and advanced deep-learning models
Source: Plant Methods. 2023 Nov 6;19:122. doi: 10.1186/s13007-023-01101-2 (PMC10629126; doi:10.1186/s13007-023-01101-2)
Supplement: Supplementary file 5 — Additional file 5: Models training plots for Zea mays (5.1) and mixed data (5.2). 2 images. [file 13007_2023_1101_MOESM5_ESM.pdf]

Additional file 5.

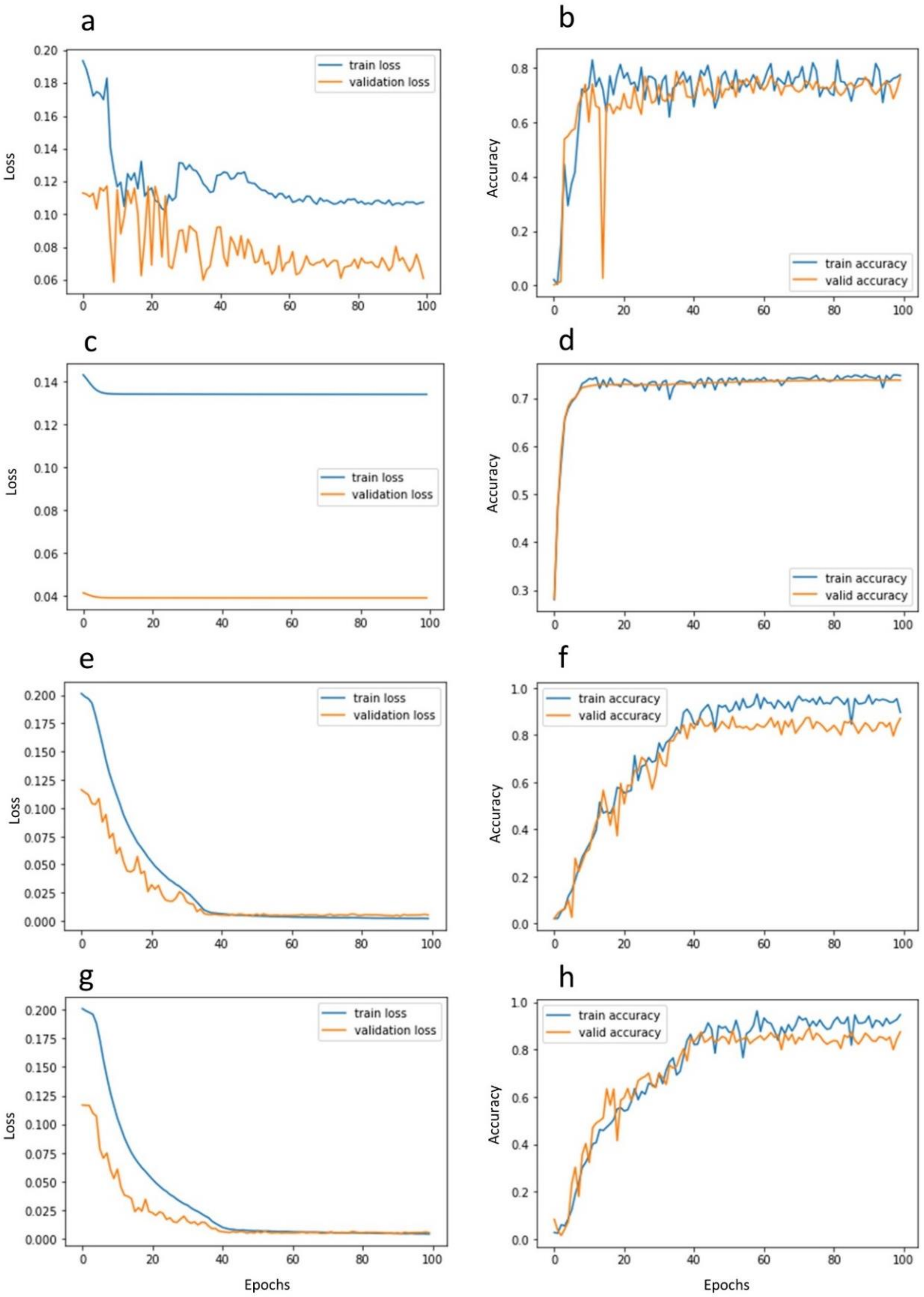

Additional file 5.1 Models training plots on *Zea mays* data (100 epochs): **a-b** SegRoot, **c-d** UNetGNRes, **e-f** U-Net SE-ResNeXt-101 (32x4d), and **g-h** U-Net EfficientNet-b6. Left column are loss plots (**a, c, e, g**), see Additional file 3 for units. Right column are accuracy plots (**b, d, f, h**); accuracy is defined as 0.7 x SSIM + 0.3 x IoU. Note: difference in y-axis label scale

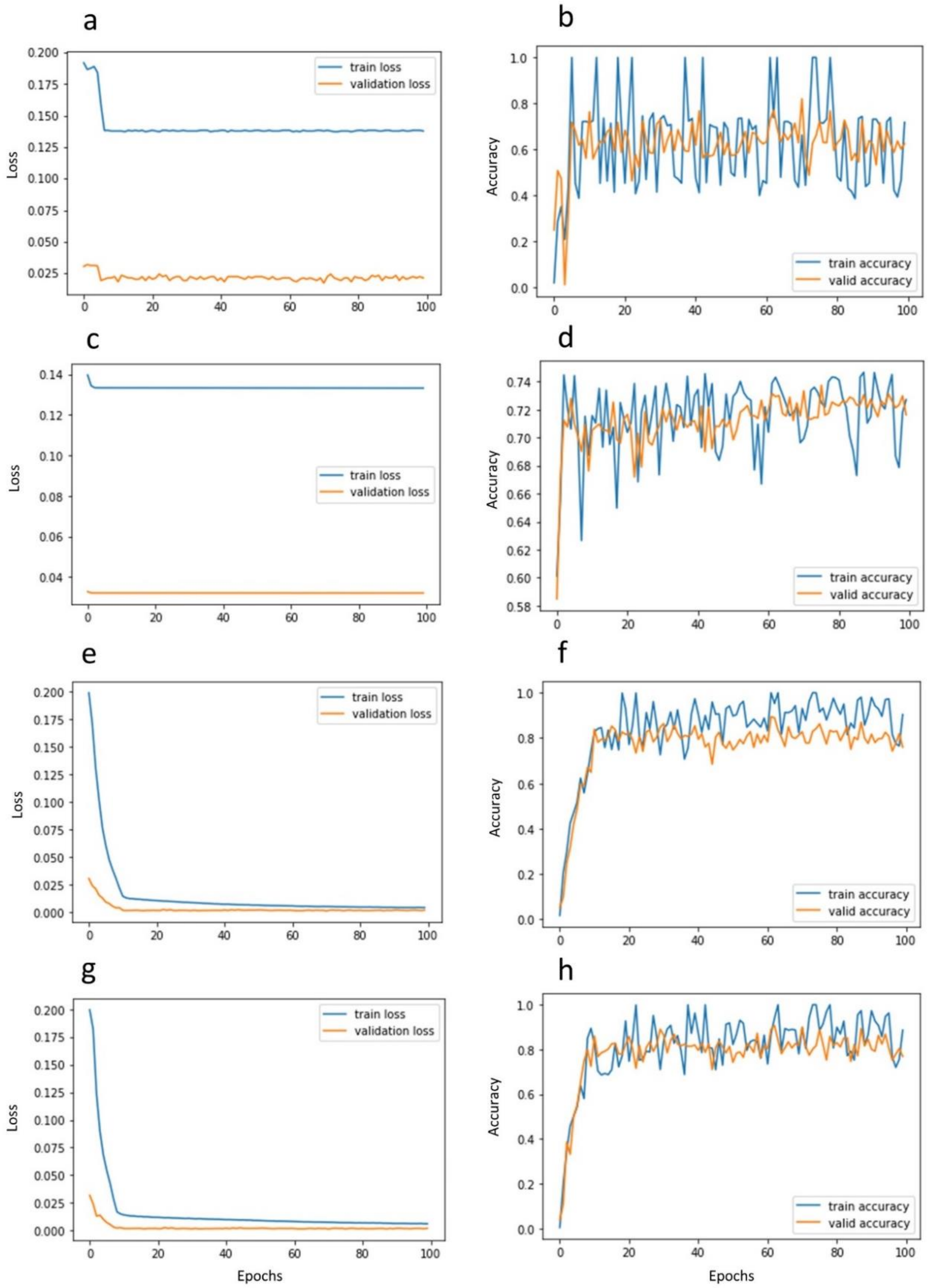

Additional file 5.2 Models training plots on mixed data (100 epochs): **a-b** SegRoot, **c-d** UNetGNRes, **e-f** U-Net SE-ResNeXt-101 (32x4d), and **g-h** U-Net EfficientNet-b6. Left column are loss plots (**a, c, e, g**), see Additional file 3 for units. Right column are accuracy plots (**b, d, f, h**); accuracy is defined as 0.7 x SSIM + 0.3 x IoU
